# Meteorological factors against COVID-19 and the role of human mobility

**DOI:** 10.1371/journal.pone.0252405

**Published:** 2021-06-04

**Authors:** Olivier Damette, Clément Mathonnat, Stéphane Goutte

**Affiliations:** 1 Faculté de Droit et de Sciences Economiques et BETA, Université de Lorraine, Lorraine, France; 2 Climate Economic Chair Paris Dauphine, France; 3 CEMOTEV, Université Versailles Saint-Quentin en Yvelines (Paris Saclay), Versailles, France; World Energy and Meteorology Council, UNITED KINGDOM

## Abstract

In the vein of recent empirical literature, we reassessed the impact of weather factors on Covid-19 daily cases and fatalities in a panel of 37 OECD countries between 1st January and 27th July 2020. We considered five different meteorological factors. For the first time, we used a dynamic panel model and considered two different kinds of channels between climate and Covid-19 virus: direct/physical factors related to the survival and durability dynamics of the virus on surfaces and outdoors and indirect/social factors through human behaviour and individual mobility, such as walking or driving outdoors, to capture the impact of weather on social distancing and, thus, on Covid-19 cases and fatalities. Our work revealed that temperature, humidity and solar radiation, which has been clearly under considered in previous studies, significantly reduce the number of Covid-19 cases and fatalities. Indirect effects through human behaviour, i.e., correlations between temperature (or solar radiation) and human mobility, were significantly positive and should be considered to correctly assess the effects of climatic factors. Increasing temperature, humidity or solar radiation effects were positively correlated with increasing mobility effects on Covid-19 cases and fatalities. The net effect from weather on the Covid-19 outbreak will, thus, be the result of the physical/direct negative effect of temperature or solar radiation and the mobility/indirect positive effect due to the interaction between human mobility and those meterological variables. Reducing direct effects of temperature and solar radiation on Covid-19 cases and fatalities, when they were significant, were partly and slightly compensated for positive indirect effects through human mobility. Suitable control policies should be implemented to control mobility and social distancing even when the weather is favourable to reduce the spread of the Covid-19 virus.

## Introduction

Faced with the global pandemic of a new coronavirus, we need to better understand the main factors that may influence the spread of the Covid-19 virus. In the Northern Hemisphere, it was widely hoped last winter that the transition to higher temperatures could slow the spread of the pandemic. This sentiment was based on the principle that the flu or influenza incidence increases during winter periods when temperature and humidity levels are likely to be at lower levels but are very moderate during summer [1]. Over the longer term, as more people develop immunity, some researchers suggested that Covid-19 may likely fall into a seasonal pattern like those seen with diseases caused by other coronaviruses. However, pandemic viruses can behave differently, as underlined for pandemic influenza, such as 2009 A/H1N1A or the Spanish flu, at least in the short-run [1]. Nonetheless, some hints from recent lab experiments or statistical studies suggest that increased temperature and humidity may reduce the viability of SARS-CoV-2.

### Lab experiments: Role of temperature and humidity

Though studies about the survival times of the Covid-19 virus are still under investigation, some lab experiments suggest that high levels of temperature and humidity can reduce the persistence and activation of Covid-19. In line with many other respiratory pathogens showing seasonality, SARS-CoV-2 might be sensitive to environmental factors, especially absolute and relative humidity conditions and temperature [2] showed that human coronaviruses can remain infectious on inanimate surfaces at room temperature for up to 9 days, but the duration of persistence is shorter at a temperature of 30°C or more. A higher temperature, such as 30 or 40°C, reduced the duration of persistence of highly pathogenic MERS-CoV, TGEV, and MHV.

Indeed, several lab experiment studies found that for other human coronaviruses, the duration of persistence is shorter in warm conditions [2] and tends to confirm the existence of seasonal patterns [3] demonstrated that the stability of HCoV-19 and SARS-CoV-1 under the experimental circumstances tested was similar [4] confirmed for the previous SARS coronavirus that virus viability was rapidly lost at higher temperatures and relative humidity levels, also leading to different epidemic curves in countries with subtropical and tropical areas, as well as considering air-conditioned environments. Finally, these results are in accordance with other previous lab experiments on different viruses, such as the gastroenteritis virus and mouse hepatitis virus [5], to determine the effects of air temperature and humidity on pathogenic viruses, such as SARS-Cov, and confirm the role of high temperatures (20 and 40°C), as well as the existence of non-monotonic relationships. Other studies [6, 7] showed that cold and dry conditions increase the transmission of the virus.

### Statistical studies: Mixed results

Recently, a few statistical studies estimated and simulated how seasonal changes in temperature might influence the trajectory of Covid-19 in cities around the world and especially in the US. A few robust studies [8–10] concluded that climate may be a marginally significant driver in evaluating the course of the Covid-19 pandemic. However, despite several new empirical investigations, the conclusions of the literature about the climate impact are still mixed (see the differences between the results obtained by [11–15] and [16–21]), and real observations do not validate previous lab projections. The negative effects of high levels of temperature and humidity found in some recent empirical studies [11–15] seem consistent with studies about the effect of physical factors on the virus and its survival rate conducted in experimental works. Regions with low humidity and average temperatures between 40 and 50°F (4 to 10°C) are likely to increase the spread of the virus [10]; above 25°F, there is a strong association between temperature and reduced transmission rates, with the largest effect a 30 to 40% reduction, but, in most locations, even 40%, which was only seen for very hot and humid conditions, still left Covid-19 climbing at an exponential rate [13, 14] showed that although cases of COVID-19 are reported all over the world, most outbreaks display a pattern of clustering in relatively cold and dry areas.

However, this positive association between climate conditions and Covid-19 is a controversial debate, and the relationship may be weak [16]. Even if one assumes that SARS-CoV-2 is as sensitive to climate as other seasonal viruses, warmer temperatures in summer still would not be enough right now to slow its rapid initial spread through the human population [17] found a positive association between daily death counts and diurnal temperature range [18] identified a positive linear relationship between Covid-19 cases and mean temperatures but found no clear evidence that the counts of Covid-19 cases were reduced when the weather was warmer [19] did not find an association between relatively high temperatures (up to 20°C) and the spread rate of the virus [20] was doubtful about the existence of a significant relationship between high absolute humidity and the survival of this new virus.

It is possible that the lack of controls included in the regression explain these different empirical results [21] showed that Covid-19 growth rates peaked in temperate regions of the Northern Hemisphere with a mean temperature of about 5°C and a specific humidity of 4–6 g/m^3^ during the outbreak period by controlling for population size, density, and health expenditure from January to March 2020.

### A lack of work about other climatic factors (solar radiation, wind speed, and precipitation)

Beyond the previous controversial conclusions, we found that there is a lack of work about other climatic factors, such as solar radiation, wind speed, and precipitation. Precipitation and wind speed generally have a positive impact on transmission rate, which could result from people spending more time indoors [10] considered wind speed (log of Km/hour), precipitation (log of millimetres), and ultraviolet index (25 milliwatts/m^2^), as well as squared ultraviolet index as potential drivers of the Covid-19 reproduction number and found a U-shaped relationship between UV index and the transmission rate. UV may help more temperate countries during the summer but increase risk in equatorial regions with very high levels of UV exposure [22–24] are in accordance for a negative association between UV levels and Covid-19 cases.

The wind speed is likely to be another determinant of Covid-19 spread since human saliva-disease-carrier droplets may travel up to unexpected considerable distances depending on the wind speed [25, 26] assumed that wind speed can affect droplet stability in the environment or the survival of viruses, like air temperature, and as a result, the transmission rate. Although wind speed is not an important factor if modelled as the only explanatory variable, it represented a necessary factor in their final model. For Turkey, [27] found that the 14-day lag of the average wind speed had the highest (positive) correlation with the number of cases. This wind speed effect was also significant for [10] and [25] but not for [28].

### Theoretical assumptions

Regarding mixed conclusions from the existing literature, the motivation and value added of our paper is to investigate the role of both direct and indirect climatic factors i.e. both physical (survival life of the virus) and human behaviors channels *via* the human displacements. Note that mobility was not related to weather in a homogeneous manner. Mobility due to leisure purposes was more impacted by climatic conditions than mobility for work purposes, for instance, that is an incompressible task *de facto* not sensitive to climatic conditions Direct climatic patterns are tested by investigating the significance of coefficients associated with five climatic factors: temperature, humidity, precipitation, wind speed, and solar radiation. Furthermore, we introduced a mobility variable to empirically test the presence of indirect effects.

Regarding the existing literature, we formulated a set of theoretical assumptions that we further empirically tested. These assumptions are summarised in Table 1.

**Table 1:** Expected direct and indirect climatic effects.

Climatic factor	Direct effect	Indirect effect
*Temperature*	**-**	**- or +**
*Precipitation*	**?**	**+**
*Humidity*	**-**	**?**
*Wind speed*	**- or +**	**?**
*Solar radiation*	**-**	**- or +**

Note: The symbol “-”denotes a negative effect i.e. a decreasing number of infected cases or fatalities due to the climatic factor, whereas the symbol “+” denotes a positive effect, i.e., an increasing number of cases or fatalities due to the climatic factor. For example, the “-”effect associated with the temperature line means that higher temperatures are likely to reduce the number of cases and fatalities. We also use the “?” symbol, denoting that there exists some uncertainty due to the absence of significant investigation about this question in the previous literature or previous mixed results.

Temperatures are expected to directly impact the virus negatively, considering lab studies. However, this effect might be rather asymmetric; only sufficiently warm temperatures are likely to hinder the survival of the virus, but the effect is less significant for cold to moderate temperatures. Indirect effects associated with temperature are difficult to evaluate. Indeed, warmer temperatures increase incentives for inhabitants to go outdoors, thus, reducing the transmission rate that is particularly high in closed areas, and low temperatures are incentives for people to stay indoors (low mobility), which is a source of increasing diffusion of the virus. Thus, it may be associated with a negative correlation coefficient. However, warmer temperatures will not systematically reduce virus transmission. In some cases, outdoor activities (in parks, shopping, meetings, etc.) could lead to a reduction of social distancing and, thus, increase the transmission rate of the virus. In addition, in times of very high temperatures, people will prefer to stay at home and use air conditioning that is likely to be bad for air quality and virus spread. Consequently, the indirect effects and the overall effect of temperature on the Covid-19 virus are very difficult to forecast.

Precipitation has no expected direct effect on Covid-19 outcomes, but indirect effects are expected; indeed, too much rain could generate incentives to stay indoors and increase the transmission of the virus. Humidity has been identified as a direct negative driver in some lab experiments, but its indirect effect through human behaviour is difficult to evaluate. The same observation stands for wind, which has sometimes been associated with a positive effect *via* droplet dynamics. Solar radiation is likely to reduce the durability of the virus (negative direct effect), and its indirect effects are expected to be relatively similar to those associated with temperature.

Therefore, we expected that the net effect of meteorological factors on Covid-19 spread will be the result of two different kinds of effects: direct and indirect. As shown in Table 1, signs from direct and indirect effects can be potentially opposed.

## Data and methods

### Data

We selected a set of 37 OECD countries over the January-September 2020 period. Our choice (see Table A in the S1 Appendix for the complete description of the countries) was motivated by 1) the availability of data and reliability, 2) a focus on a relatively homogeneous sample of developed countries with comparable standards of life and climate regimes to better identify the effect of meteorological factors, 3) most selected OECD countries in our sample encountered the first wave of the pandemic at the same time (see S1 Appendix part A), and 4) considering data between January and the end of July (September in robustness) was enough to cover the first wave; since the second wave of the pandemic is in progress, we decided not to consider data after September. In conclusion, from an econometric point of view, we obtained a panel dataset with 37 cross-section units (countries) and more than 6,400 observations.

### Covid-19 data

We obtained the number of confirmed Covid-19 cases and deaths for the countries in our sample from multiple sources through the DELVE initiative between 1st January 2020 and 27th July 2020 and estimated the population in 2020 from the World Bank’s World Development Indicators database. Covid-19 data was aggregated by [29], DELVE Global COVID-19 Dataset and are available at http://rs-delve.github.io/data\_\software/global-dataset.html.

Previous academic research commonly used infected cases, fatality rate, or both as endogenous variables. Here, for comparison purposes with previous studies we used both indicators although infected case counts can be biased. Recently, [30] considered missing data on tests for infection issues, as well as imperfect accuracy of tests and showed that the infection rate might be substantially higher or lower than reported; for instance, Illinois, New York, and Italy infection rates are substantially lower than reported. In some countries, during pandemic waves, testing has been stopped due to lack of time concerns. Another reason to consider both infected cases and fatality rates is that these two variables, though related, do not capture the same Covid-19 outcomes (short-run versus long-run dynamics). Infected people are generally officially considered infected (by testing) only a few days (between 7 and 14 days) after the contamination day, although the median incubation is estimated to be 5 days [31]. Thus, infected case counts capture a short-run effect of potential climatic or social distancing variables on the Covid-19 pandemic. However, the delay is longer for death counts considering the time people can develop the disease and stay in hospitals. Therefore, the fatality rate captures a long-run dimension of the transmission from climatic and other determinants (mitigation policies for example) to Covid-19 outcome.

We considered the presence of many zero values for the infected cases and fatality rate (especially for the latter) in the beginning of the sample range (January essentially), since this can lead to biased estimates. Thus, we chose a restrictive (less longer) sample as a benchmark: February 1st was assumed as a starting time for the pandemic (infected cases) for all countries, whereas February 15th was chosen for fatality counts. We considered epidemiological and statistical arguments when we selected these particular starting dates. Considering these time windows enabled us to start our estimates when the first significant number of Covid-19 cases and deaths was observed, as well as to have enough observations in the time dimension to perform a dynamic model. In the robustness tests part, we relaxed this assumption and considered the totality of the available data. An alternative solution would be to build a normalized sample, where time t = 1 is set when a country reports at least 1000 infected people, for instance. Robustness checks showed that the heterogeneity of the starting dates was not a significant issue.

### Meteorological data

We obtained daily meteorological conditions in the selected countries for the entire period from UK met using impressive data computation from the DELVE program [29]. The definition of the five climatic factors is summarised in Table 2. For each country, all climatic observations were weighted by the population of the cities selected to compute the aggregate series. Furthermore, the five meteorological factors were standardized for comparison purposes and coefficient scale homogeneity [(current value–mean)/standard deviation]; thus, it was possible to compare the effect of one unit of temperature with one of precipitation.

**Table 2:** Climatic variables.

Variable	Definition	Unit
Temperature	Average daily mean of temperatures	Celcius Degrees
Humidity	Average daily humidity of air	Kilograms of water vapour per kilogram
Wind speed	Average daily wind speed	Meters per second
Solar radiation	Average daily short-wave radiation	W/m^2 (Watts per square meter)
Precipitation	Average daily precipitation	mm / hr

### Mobility data

Finally, to proxy human mobility, we used Apple mobility reports data available at: https://www.apple.com/covid19/mobility. Two main variables were used to proxy mobility, namely *driving* and *walking*. We focused primarily on the driving variable but also used walking as a robustness check. These data were indices (100 basis) and computed as the percentage of change in routing requests since 13th January 2020. Note that Google mobility reports data were also been considered as an alternative mobility dataset and exhibited very similar trends (statistical analysis available upon request).

The following Table 3 presents the usual descriptive statistics for the three types of variables (Covid-19 outcomes, Climatic factors, Mobility).

**Table 3:** Descriptive statistics.

Variable	Obs.	Mean	Std. Dev.	Min	Max
casepop	6,586	2.09	4.09	0	37.38
deathpop	6,068	0.13	0.30	0	5.53
Temperature	6,438	0.14	0.95	-3.35	2.66
Solar radiation	6,438	0.19	0.89	-1.86	2.07
Precipitation	6,438	0.01	1.01	-0.59	15.13
Humidity	6,438	0.09	1.00	-1.85	3.51
Wind speed	6,438	-0.02	0.97	-1.56	5.96
Mobility (driving)	6,512	96.64	46.09	10.93	340.21

Note: climatic variables are standardized one.

## Econometric framework

### A dynamic panel data model

Even though there are significant differences in meteorological conditions between our set of countries, they were all located in a relatively homogeneous North Hemisphere (except Australia, Chile and New Zealand). Thus, all conditions worked with a suitable econometric panel; unobserved heterogeneity across countries was included inside a relative homogeneous panel [see the "to pool or not pool debate" developed by Pesaran et al. [32]].

Before estimating our model, we proceeded to the usual panel unit root tests to evaluate the properties of our series. Maddala Wu and Pesaran CIPS tests were performed and showed that the dynamics of the series were driven by deterministic and stationary stochastic processes. Results favoured the stationarity of our series and were more clear-cut (at a 5% confidence level) in the case of the Pesaran CIPS test, which is more relevant since it considers cross-section dependence under the null hypothesis. All tests are available upon request.

Our baseline model was written in a dynamic panel form as follows:

$$y_{it}=\alpha_{0}+\underset{pandemic\;dynamic\;effect}{\underbrace{\alpha_{1}y_{it-1}+\alpha_{2}y_{it-k}}}+\underset{direct\;climate\;effect}{\underbrace{\alpha_{3}C_{it-p}}}+\mu_{i}+\delta_{t}+\in_{it}$$ (1)

where the subscripts *i* and *t* represent country index and periods (days), respectively. The dependent variable, *y*_*it*_, is the number of infected people (*casespop*) or deaths (*deathpop*) per capita (considering the population size) at time *t*. Thus, *y*_*it*−1_, for instance, is an autoregressive term of order one that accounts for the persistent feature of the pandemic (effect of the previous period on the current situation). *C*_*it*−*p*_ is a vector of variables depicting the effects of meteorological conditions in day *t-p*. Country-specific fixed effect, *μ*_*i*_, was included to control for time-invariant omitted-variable bias and ∈_*it*_ is the error term. *δ*_*t*_ is a deterministic time trend (squared trend was also tested) that controls the deterministic dynamics of the epidemics over the studied period and captures some unobserved information about the pandemic common to all countries (changes in human behaviours, or public mitigation policies such as testing or tracking for instance). In addition, lagged terms *y*_*it*−*k*_ capture the stochastic part of the pandemic dynamics. We assumed *k* was equal to 7 or 14 lags/days in our baseline specifications, considering incubation and confirmation periods presented in the Covid-19 literature. Moreover, for logical reasons, since climatic factors do not immediately impact Covid-19 spread, the climatic variables were also included in our model with a lag of order *p*. Indeed, there were delays between the time of potential infection corresponding to certain climatic events and the time of official counting of a potentially infected person (or fatality). Therefore, when dealing with *p*, 7 or 14 days were considered when *casepop* was used as an endogenous variable, considering the short period between transmission and infection.

A 14 day/lag delay was considered a benchmark when dealing with *deathpop* because of the more important lag length assumed between infection and death related to Covid-19. More lags (28 = one month or 42) were also considered in robustness checks to consider the dynamic persistence of the pandemic. Again, *casepop* and *deathpop* give a short-run and medium/long-run time perspective, respectively, of the dramatic outcomes of the pandemic. Notably, when *y*_*it*_ is the fatality rate, we also added the ratio of infected cases per capita in our benchmark specification to account for the fact that the level of the pandemic can impact the fatality rate. The reason behind this is to control for a level effect and a kind of saturation effect of the health system (too many infected people to manage is likely to increase the fatality rate).

We developed an extended specification incorporating mobility indices to investigate potential indirect effects of meteorological factors *via* the mobility and thus the impact of climate on human behaviour (interacted variable). Thus, Eq (1) becomes Eq (2) with *M* as the mobility (driving) index:

$$y_{it}=\alpha_{0}+\underset{pandemic\;dynamic\;effect}{\underbrace{\alpha_{1}y_{it-1}+\alpha_{2}y_{it-k}}}+\underset{direct\;climate\;effect}{\underbrace{\alpha_{3}C_{it-p}}}+\underset{mobility\;effect}{\underbrace{\alpha_{4}M_{it-p}}}+\underset{indirect\;mobility\;effect}{\underbrace{\alpha_{5}C_{it-p}*M_{it-p}}}+\mu_{i}+\delta_{t}+\in_{it}$$ (2)

Hence, we controlled the direct effect of climatic factor *α*_3_, which can be decomposed in a direct effect in the absence of individual mobility (*α*_4_ is thus equal to zero) and a residual effect influenced by positive mobility (*α*_5_). In other words, we now estimated the effects of meteorological factors conditionally on mobility dynamics.

Eqs 1 and 2 were estimated by several estimators and especially by the mean group (MG) model of Pesaran and Smith [33] and the dynamic fixed effect (DFE) estimator that was relevant for macro panels, such as the one used in this paper, when the time dimension exceeds the cross-section dimension. Indeed, in our sample, the mean of the time dimension noted *T* is equal to 174 and was, thus, largely superior to the cross section dimension noted *N*, which is equal to 37. The MG estimator consisted of estimating each regression separately for each panel member *i* (country here) with a minimum of econometric restrictions. All estimated coefficients were heterogeneous and subsequently averaged across countries *via* a simple unweighted average. An intercept was included to capture country fixed effects, as well as a linear trend. Considering the minimal number of restrictions of this estimator, a large time-series dimension was needed to respect consistency. A small *N* (inferior to 20) was also a problem, as it increased the sensitivity to outliers, but we selected 37 OECD countries here. The high dimension of our panel allowed the use of this estimator in this study. In contrast, the Dynamic Fixed Effect estimator (DFE), more similar to the PMG estimator developed by [34], assumes the slope coefficients to be equal both in the long- and short-run.

### Identification issues

Although we applied appropriate macro-panel estimators to our data, several issues, nonetheless, emerged. First, using dynamic models, we were vulnerable to the so-called Nickel (1981) bias. Here, this bias was relatively negligible, notably, considering the important time length of our series. Second, panel regressions may be exposed to an omitted variables bias. It would be possible to include control variables, such as control measures (e.g., testing, mask wearing, travel controls) or structural determinants (e.g., population density and demographics, such as the population over 65 age, tourist flows, GDP per capita, and measures of health infrastructures).

Considering the so-called problem of controls in the ‘climate macroeconomy literature’, our set of explanatory variables was assumed to be restricted to climatic variables, mobility and deterministic trend to avoid an over-controlling problem [35]. In addition, considering data availability and the fact that, in our sample based on daily Covid-19 cases and fatalities, they are time-invariant variables (GDP per capita, CO2 emissions, or the part of the population over 65 age for instance), we captured these unobservable variables via the lagged term *y*_*it*−1_ in addition to country fixed effects. Another identification issue was related to the potential reverse causality bias related to our Covid-19 variables: news about contemporaneous dynamics of the Covid-19 outbreaks and counts can change human behaviour in real time and social distancing. Therefore, lags of dependent variables were added to our model. Finally, persistence and multicollinearity are other usual issues in panel studies. We controlled for both by computing autocorrelations LM (Lagrange Multiplier) tests and VIF/Tolerance ratios after each estimated regression. In the robustness section, we also considered endogeneity issues about the mobility index variable and other tests related to the specification of our econometric model, the choice of an alternative estimator, and several changes in the sample composition.

Stata Software was used to compute all estimates and we referred to the usual confidence levels at 1, 5, and 10% to assess the significance of our coefficients. To derive robust conclusions, 5% and 1% levels were considered.

## Results

### Direct effect

Our baseline results are reported in Tables 4 and 5. The five meteorological factors were standardized (centred-reduced variables; note that results associated with non-standardized variables lead to very similar conclusions, see Tables I1-I4 in S1 Appendix) for comparison purposes and coefficient scale homogeneity. We presented 10 columns or specifications; each pair of successive columns is associated with the same climatic factor (columns 1 and 2 for temperature, columns 3 and 4 for humidity, etc.). Thus, considering the high correlation between them, we focused on a single specific climatic factor (going from temperature to wind speed) and evaluated its effects with two different estimators, DFE and MG; for example, it is likely that high temperatures occurred at high solar radiation levels with very low precipitation levels and low wind speed.

**Table 4:** Direct effects of climate variables on Covid-19 infected cases.

	Covid-19 infected cases									
	DFE	MG	DFE	MG	DFE	MG	DFE	MG	DFE	MG
	(1)	(2)	(3)	(4)	(5)	(6)	(7)	(8)	(9)	(10)
casepop (t-1)	0.700***	0.618***	0.699***	0.620***	0.702***	0.618***	0.702***	0.629***	0.702***	0.629***
	[0.0357]	[0.0340]	[0.0359]	[0.0336]	[0.0356]	[0.0342]	[0.0357]	[0.0333]	[0.0356]	[0.0330]
casepop (t-7)	0.242***	0.255***	0.246***	0.257***	0.244***	0.253***	0.244***	0.256***	0.244***	0.256***
	[0.0355]	[0.0299]	[0.0359]	[0.0298]	[0.0357]	[0.0296]	[0.0356]	[0.0298]	[0.0356]	[0.0299]
temperature (t-7)	-0.103**	-0.209**								
	[0.0493]	[0.0858]								
solar radiation (t-7)			-0.111**	-0.106*						
			[0.0430]	[0.0627]						
humidity (t-7)					-0.00482	-0.0772				
					[0.0419]	[0.0615]				
precipitation (t-7)							-0.0184	-0.0370*		
							[0.0125]	[0.0193]		
wind speed (t-7)									-0.0282*	-0.110*
									[0.0156]	[0.0604]
Trend	Yes	Yes	Yes	Yes	Yes	Yes	Yes	Yes	Yes	Yes
Observations	6,438	6,438	6,438	6,438	6,438	6,438	6,438	6,438	6,438	6,438
Country	37	37	37	37	37	37	37	37	37	37
R-squared	0.821		0.821		0.821		0.821		0.821	

Note: the coefficients displayed are marginal effects. Standard errors (robust to within-country correlations for DFE) are reported in brackets.

*** p < 0.01,

** p < 0.05,

* p < 0.1.

**Table 5:** Direct effects of climate variables on Covid-19 fatalities.

	Covid-19 fatalities									
	DFE	MG	DFE	MG	DFE	MG	DFE	MG	DFE	MG
	(1)	(2)	(3)	(4)	(5)	(6)	(7)	(8)	(9)	(10)
deathpop (t-1)	0.590***	0.365***	0.593***	0.367***	0.590***	0.359***	0.593***	0.373***	0.593***	0.369***
	[0.111]	[0.0482]	[0.111]	[0.0484]	[0.112]	[0.0486]	[0.111]	[0.0480]	[0.111]	[0.0490]
deathpop (t-14)	0.155***	0.107***	0.154***	0.102***	0.152***	0.0974***	0.153***	0.102***	0.152***	0.103***
	[0.0468]	[0.0254]	[0.0477]	[0.0253]	[0.0460]	[0.0254]	[0.0468]	[0.0251]	[0.0463]	[0.0251]
casepop (t-14)	0.00806**	0.0185***	0.00857**	0.0187***	0.00832**	0.0181***	0.00865**	0.0185***	0.00868**	0.0188***
	[0.00327]	[0.00409]	[0.00341]	[0.00421]	[0.00331]	[0.00405]	[0.00344]	[0.00417]	[0.00344]	[0.00417]
temperature (t-14)	-0.0178***	-0.0167**								
	[0.00533]	[0.00745]								
solar radiation (t-14)			-0.00296	0.00823						
			[0.00385]	[0.00552]						
humidity (t-14)					-0.0143**	-0.0195***				
					[0.00529]	[0.00562]				
precipitation (t-14)							0.000349	0.000249		
							[0.00274]	[0.00386]		
wind speed (t-14)									-0.00305	-0.00377
									[0.00285]	[0.00903]
Trend	Yes	Yes	Yes	Yes	Yes	Yes	Yes	Yes	Yes	Yes
Observations	5,920	5,920	5,920	5,920	5,920	5,920	5,920	5,920	5,920	5,920
Country	37	37	37	37	37	37	37	37	37	37
R-squared	0.563		0.562		0.563		0.562		0.562	

Note: the coefficients displayed are marginal effects. Standard errors (robust to within-country correlations for DFE) are reported in brackets.

*** p < 0.01,

** p < 0.05,

* p < 0.1.

Although we computed many different models, we only presented here the most reliable results based on epidemiological concerns; indeed, we considered the 7-day lag of climate variables for case counts (incubation period). When dealing with the fatality ratio, more delays in the effect of climatic variables were considered, as we included the 14-day lag of these variables. Thus, our main objective was to give a short-run and a long-run point of view on the epidemic through these two different outcomes variables.

**Fig 1:**
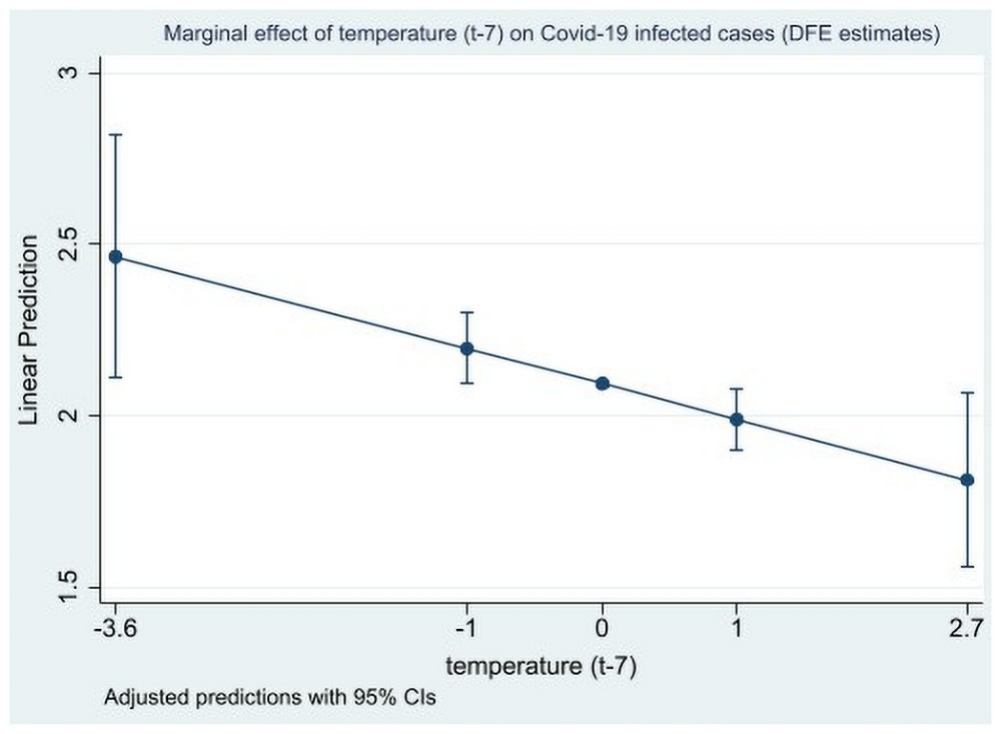
Marginal effects of temperature (t-7) on Covid-19 infected cases (direct effect with DFE estimates). Marginal effects are computed at the min, -1 std. deviation, mean, + 1 std. deviation, and max values of the (standardized) temperature variable.

**Fig 2:**
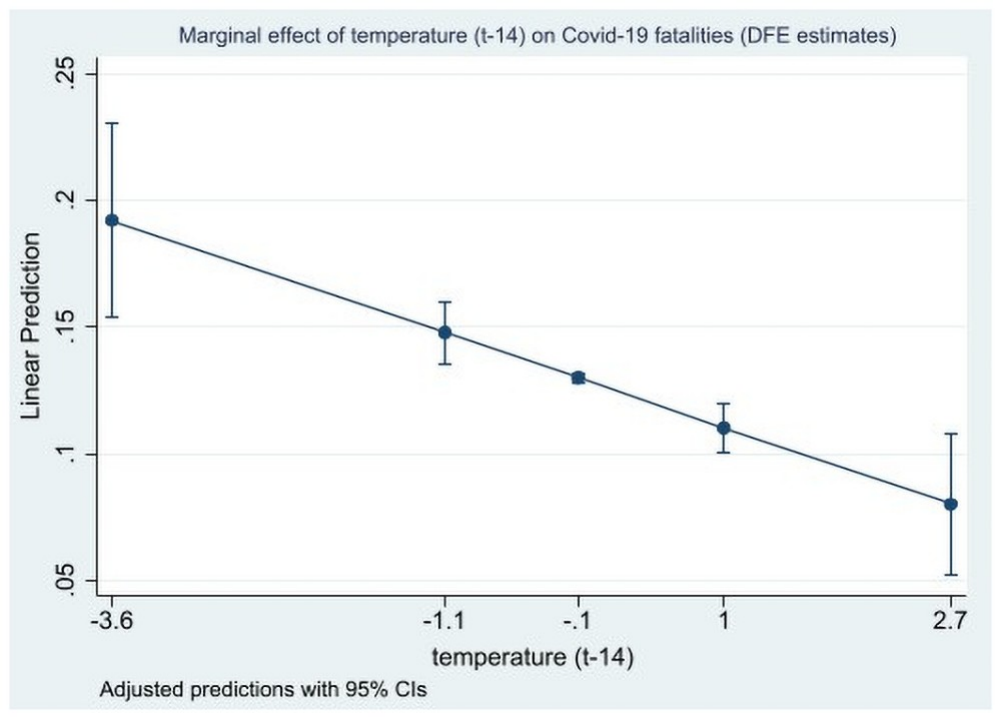
Marginal effects of temperature (t-14) on Covid-19 fatalities (direct effect with DFE estimates). Marginal effects are computed at the min, -1 std. deviation, mean, + 1 std. deviation, and max values of the (standardized) temperature variable.

The results in Tables 4 and 5 show that temperature and humidity levels (only for 14 days/lags), the most studied factor in the previous literature, had a significant negative effect on the Covid-19 infected cases’ ratio and on fatality rates. These results are also illustrated by the marginal effects plotted on Figs 1 and 2. Consequently, these two factors are likely to reduce the spread of the virus through a direct effect, as suggested by much of the previous literature. Qualitatively, the same results were derived for wind speed and solar radiation, and to a lesser extent for precipitation, that have been less investigated in previous literature. The results were robust to lag and estimator choices. Therefore, these first estimates tend to corroborate the conclusions of a part of the previous statistical literature about a small but significant effect of the climatic factors—mainly temperature but not only—as a reducing driver of the Covid-19 outbreak, see for instance [10].

### Indirect effects

In previous analyses, the coefficients associated with meteorological factors were likely to be biased if any effects from other variables were significantly different from zero, especially *via* human mobility. Mobility can impact Covid-19 transmission rates, as demonstrated by [10] and [36]. Thus, we evaluated both direct and indirect effects *via* interacted terms by introducing a mobility variable. Direct or physical effects refer to the impact of higher temperatures, humidity, or solar radiation, for instance, on the survival of the virus onto surfaces or as droplets. This decomposition of the effects enabled us to investigate if the climatic factors may impact Covid-19 outcomes conditionally to the level of displacements of the population (here, driving, but our results are robust when *walking* is used instead). The interaction term solar radiation*mobility was significantly positive at a high level of confidence concerning infected case specifications (Table 6). In other words, during sunshine periods, mobility and climatic effects were positively correlated and tended to reinforce themselves within a seven-day action period. The same results were derived for temperature and humidity but in a less clear manner (only at 10% level for some specifications). In addition, these results were more significant when considering the fatality rate as a dependent variable (Table 7). This could be explained by the higher relevance of the fatality rate variable in comparison to the infected cases used to capture the true dynamics of pandemics.

**Table 6:** Indirect effects of climate variables on Covid-19 infected cases through human mobility.

	Covid-19 infected cases									
	DFE	MG	DFE	MG	DFE	MG	DFE	MG	DFE	MG
	(1)	(2)	(3)	(4)	(5)	(6)	(7)	(8)	(9)	(10)
casepop (t-1)	0.697***	0.521***	0.693***	0.548***	0.700***	0.533***	0.701***	0.583***	0.702***	0.583***
	[0.0357]	[0.0406]	[0.0361]	[0.0361]	[0.0352]	[0.0411]	[0.0355]	[0.0361]	[0.0352]	[0.0362]
casepop (t-7)	0.240***	0.223***	0.243***	0.244***	0.241***	0.219***	0.244***	0.235***	0.244***	0.240***
	[0.0360]	[0.0319]	[0.0363]	[0.0312]	[0.0361]	[0.0309]	[0.0361]	[0.0304]	[0.0360]	[0.0301]
mobility (t-7)	-0.000308	-0.00259	-0.00157*	-0.00528***	-0.000420	-0.00494***	-8.33e-05	-0.00300**	-7.67e-05	-0.00275**
	[0.000664]	[0.00365]	[0.000789]	[0.00144]	[0.000523]	[0.00133]	[0.000496]	[0.00128]	[0.000550]	[0.00128]
temperature (t-7)	-0.280***	-0.732***								
	[0.0966]	[0.225]								
temperature*mobility (t-7)	0.00168*	0.00568*								
	[0.000982]	[0.00329]								
solar radiation (t-7)			-0.397***	-0.650***						
			[0.134]	[0.201]						
solar radiation*mobility (t-7)			0.00282**	0.00664***						
			[0.00112]	[0.00207]						
humidity (t-7)					-0.134*	-0.713***				
					[0.0682]	[0.141]				
humidity*mobility (t-7)					0.00130*	0.00712***				
					[0.000670]	[0.00140]				
precipitation (t-7)							-0.0807	-0.0751		
							[0.0502]	[0.0529]		
precipitation*mobility (t-7)							0.000660	0.000242		
							[0.000465]	[0.000569]		
wind speed (t-7)									-0.0503	0.0514
									[0.0799]	[0.139]
wind speed*mobility (t-7)									0.000239	-0.00156
									[0.000689]	[0.00159]
Trend	Yes	Yes	Yes	Yes	Yes	Yes	Yes	Yes	Yes	Yes
Observations	6,364	6,364	6,364	6,364	6,364	6,364	6,364	6,364	6,364	6,364
Country	37	37	37	37	37	37	37	37	37	37
R-squared	0.821		0.822		0.821		0.821		0.821	

Note: the coefficients displayed are marginal effects. Standard errors (robust to within-country correlations for DFE) are reported in brackets.

*** p < 0.01,

** p < 0.05,

* p < 0.1.

**Table 7:** Indirect effects of climate variables on Covid-19 fatalities through human mobility.

	Covid-19 fatalities									
	DFE	MG	DFE	MG	DFE	MG	DFE	MG	DFE	MG
	(1)	(2)	(3)	(4)	(5)	(6)	(7)	(8)	(9)	(10)
deathpop (t-1)	0.601***	0.255***	0.603***	0.276***	0.602***	0.256***	0.612***	0.290***	0.610***	0.287***
	[0.120]	[0.0446]	[0.121]	[0.0453]	[0.120]	[0.0453]	[0.120]	[0.0464]	[0.121]	[0.0492]
deathpop (t-14)	0.144***	0.0819***	0.142***	0.0683**	0.137***	0.0727***	0.131**	0.0550**	0.131**	0.0552**
	[0.0500]	[0.0267]	[0.0510]	[0.0272]	[0.0492]	[0.0269]	[0.0496]	[0.0273]	[0.0492]	[0.0270]
casepop (t-14)	0.00523*	0.0116***	0.00584**	0.0144***	0.00574*	0.0113***	0.00701**	0.0146***	0.00698**	0.0148***
	[0.00292]	[0.00366]	[0.00274]	[0.00418]	[0.00300]	[0.00361]	[0.00301]	[0.00413]	[0.00308]	[0.00425]
mobility (t-14)	-0.000540**	-0.00112***	-0.000671***	-0.00118***	-0.000500**	-0.00105***	-0.000450**	-0.000826***	-0.000459***	-0.000798***
	[0.000205]	[0.000292]	[0.000240]	[0.000269]	[0.000184]	[0.000225]	[0.000168]	[0.000310]	[0.000169]	[0.000285]
temperature (t-14)	-0.0620***	-0.0896***								
	[0.0192]	[0.0259]								
temperature*mobility (t-14)	0.000508***	0.000936***								
	[0.000178]	[0.000270]								
solar radiation (t-14)			-0.0606***	-0.0554***						
			[0.0204]	[0.0190]						
solar radiation*mobility (t-14)			0.000560***	0.000777***						
			[0.000200]	[0.000262]						
humidity (t-14)					-0.0512***	-0.0935***				
					[0.0171]	[0.0229]				
humidity*mobility (t-14)					0.000452***	0.000890***				
					[0.000155]	[0.000220]				
precipitation (t-14)							0.00173	-0.0267		
							[0.00955]	[0.0168]		
precipitation*mobility (t-14)							1.43e-06	0.000478		
							[7.44e-05]	[0.000386]		
wind speed (t-14)									0.00608	-0.00806
									[0.00954]	[0.0202]
wind speed*mobility (t-14)									-0.000105	6.80e-05
									[9.06e-05]	[0.000179]
Trend	Yes	Yes	Yes	Yes	Yes	Yes	Yes	Yes	Yes	Yes
Observations	5,846	5,846	5,846	5,846	5,846	5,846	5,846	5,846	5,846	5,846
Country	37	37	37	37	37	37	37	37	37	37
R-squared	0.600		0.600		0.599		0.597		0.597	

Note: the coefficients displayed are marginal effects. Standard errors (robust to within-country correlations for DFE) are reported in brackets.

*** p < 0.01,

** p < 0.05,

* p < 0.1.

We found that solar radiation always has a negative direct coefficient as it is expected to physically reduce the resistance of the virus. The interaction terms between solar radiation and mobility, as well as temperature and mobility and humidity and mobility, were positive and show that mobility and climatic variables strengthen each other. Since we focused on climatic variables, we interpreted the positive interaction term as a counterbalancing effect that was probably underestimated in previous studies. The overall (net) effect of meteorological factors was thus composed of a direct negative effect and an indirect positive effect. This net effect was negative in the short-run (at least), but our results showed that the direct physical effect of temperature, humidity, and solar radiation was partly counterbalanced by changes in human behaviour. The potential reduction of climatic factors was indeed partially and slightly compensated for by a significant positive effect stemming from the interaction between climatic variables, namely temperature, humidity, and solar radiation, and the mobility variable on Covid-19 infected cases ratio and fatality rates.

We found that the mobility *driving* variable had a direct negative influence on Covid-19 outcomes in the short-run (7 or 14 lags), see Tables 6 and 7, but a positive influence in the long-run, see Tables D1-D4 in the S1 Appendix. We interpreted this direct effect as a lockdown effect in the short-run. Indeed, most countries in our sample implemented a lockdown policy; in the short-run, the effect of this policy was influenced by reverse causal endogeneity issues, where people were asked to reduce their mobility (driving or walking, for instance) due to the strong diffusion of the virus. For robustness check purpose, we accounted for the effect of the lockdown policies, see the estimates displayed in the Tables H1, H2 in S1 Appendix, the lockdown is proxied by a dummy variable (*Dummy_lockdown*) and in Tables H3, H4 in S1 Appendix by a continuous variable computing the time between the starting time of the lockdown and the current period (*Time_lockdown*).

An increased number of cases was, thus, associated with reduced mobility. We controlled this potential endogeneity issue, and our tests revealed that when the number of lags increased (Tables D1-D4 in S1 Appendix), the sign of the mobility variable changes to be positive in the infected cases’ regression. In the long-run, increasing mobility was likely to increase transmission rates and create new clusters. In other words, increasing individual mobility was a factor of virus spread when more people were more mobile. Social distancing was likely to be reduced and the transmission rate to increase. Consequently, in the long-run, increasing mobility would increase the fatality rate of the virus, confirming that lockdown policies have probably been relevant to try to reduce the severity of the pandemic by reducing mobility.

## Robustness

### Testing for threshold effects in climate variables

Our baseline specifications assumed that the statistical relationship between meteorological factors and Covid-19 outcomes was linear. Here, we investigated the existence of threshold effects by testing quadratic functions, i.e., whether the effect of climate on the number of infected cases or the fatality rate was nonlinear. Indeed, as shown, for instance, by [10], among others, temperature (when surpassing 25°C, for example) or ultraviolet levels were likely to exhibit nonlinear patterns. Overall, our results, reported in Tables B1 and B2 in S1 Appendix suggested no threshold evidence except for the solar radiation variable when considering both the number of infected cases and the fatality rate, and for temperature and humidity only when considering DFE estimates for the number of infected cases. Thus, these results may suggest a potential inverted U-shaped relationship between solar radiation and the dynamic of Covid-19 epidemic (*casepop and deathpop)*, meaning that solar radiation would be able to reduce the number of infected people and the fatality rate only for high levels of solar radiation.

### Testing for interactions between climate variables

We tested potential interactions between climate variables that could bias the results. We focused on potential links between temperature, solar radiation, and humidity, which were the most meteorologically relevant relationships. Tables C1 and C2 in S1 Appendix show that when we considered interaction effects between these three meteorological variables, the results did not reveal clear significant interaction patterns between them. In addition, the negative direct effects of temperature, solar radiation, and humidity were significant only for some specifications.

### Increasing lags for Covid-19, climate, and human mobility variables

When we augmented the number of lags, we implicitly increased the delayed effects from climate to Covid-19 outcomes. Consequently, we controlled for the dynamics of the epidemic and considered the fact, especially for the fatality rate, that the impact of climatic factors had a very delayed origin; a huge meeting, concert, or social event could produce some dramatic effects only three or four weeks later and might result in so-called Covid-19 clusters. In addition, more lags allowed better consideration of count biases, as we previously discussed. For instance, count delay has sometimes been estimated to be 10 days (between real infection and official recording in some countries). Therefore, the delay between infection and potential disease is uncertain; most medical studies report a median incubation period for Covid-19 of approximately 5 days as shown by [30], but considering the consolidation period, 14 or 28 days are alternatives.

In Tables D1-D4 in S1 Appendix, we present increasing lag regressions. Twenty-one lags were chosen for *casepop* and 28 lags were considered for *deathpop*, corresponding to a one-month period between real infection and deaths recorded in this case. Longer periods, until two months, were also considered. Although our sample had an important time dimension, it was difficult to go beyond this lag to get robust econometric estimations. Table D3 in S1 Appendix outlines that for some estimates the mobility variable can have a direct significant positive effect on Covid-19 outcomes when a longer horizon is considered (here, 21 lags for the *casepop* variable). The interaction terms between solar radiation and mobility was still positive when considering MG estimates for infected cases in Table D3 in S1 Appendix, showing, once again, the existence of a mobility indirect effect through this climate variable. The quality of the estimates was better for the fatality rate in Table D4 in S1 Appendix with a one-month (28 lags) delay considered. Indeed, in this case, the interaction between solar radiation and mobility is significant and positive for both DFE and MG estimates, while the interaction between temperature and mobility is significant and positive only for DFE estimates. In addition, results displayed in Tables D3 and D4 in S1 Appendix show that temperature and solar radiation had a negative and significant direct effect that was slightly counterbalanced by an indirect mobility effect in line with our baseline estimates.

Overall, these results tended to shade the conclusions of the previous literature about the significant effects of climatic variables, especially for the humidity index, and showed that contrasting conclusions obtained thus far in the literature could be explained by the lags and time horizon chosen to study the effects of climatic variables on Covid-19 outcomes. A well understanding of the role of climatic factors on Covid-19 implies to go beyond simple static correlations and regressions. Finally, it was crucial to consider the short and long-run dynamics of the epidemic, and the choice of different Covid-19 outcomes as endogenous variables was not neutral.

### Endogeneity issues: System-GMM estimates

In Tables E1 and E2 in S1 Appendix, we controlled for the potential endogeneity of lagged variables, Covid-19 variables, and mobility by performing System GMM estimates. The following Syst-GMM estimates were implemented by assuming the potential endogeneity of *casepop t-7*, *casepop t-14*, *deathpop t-14*, *mobility t-7*, and *mobility t-14*. Thus, we considered the potential endogeneity of both Covid-19 and mobility. The results corroborated our previous findings about the direct and indirect effects of climatic factors on both infected cases and fatalities for temperature, solar radiation, and humidity. In most cases, classical Syst-GMM tests [AR(1), AR(2), and Hansen tests] confirmed the validity of our different sets of instruments.

### Alternative starting period

We previously assumed that samples started on 1st February 2020 for infected cases and 15th February, for the fatality rate. In this subsection, we re-estimated all regressions by considering the entire sample range and not only periods since 1st February 2020 for infected cases and from 15th February for the fatality rate. Thus, estimates were computed with data starting on 1st January 2020 for all countries. The resulting new sample was then marginally augmented. Tables F1, F2 in S1 Appendix (direct effects) and F3-F4 (both direct and indirect effects) indicated no significant differences from previous key estimates. Therefore, our results were robust to time span changes concerning the beginning period. The relatively low incidence of Covid-19 from January and February 2020 did not lead to a bias in the estimate.

### Accounting for seasonality in the climate variables

We showed in Tables J1-J8 in S1 Appendix that our results–both direct and indirect effects of climate variables on Covid-19 cases and fatalities–are robust when we include some additional variables capturing the seasonality in the climate variables. In Tables J1-J4 in S1 Appendix, we added a *season index* as a qualitative variable accounting for meteorological seasons in the Northern and Southern hemispheres: the index takes the value 1 for winter, 2 for spring, 3 for summer and 4 for autumn. Meteorological seasons have been considered under the constraint of matching our sample: for instance, the *season index* variable takes the value 1 (winter) for observations between January 1^st^ and 29^th^ February, 2020 in the Northern hemisphere and for observations between 1^st^ June and 27^th^ July, 2020 in the Southern hemisphere. Tables J1-J4 in S1 Appendix reveal that the coefficients associated with the *season index* variable are positive and significant, while the coefficients associated with climatic variables are unchanged, especially when the human mobility variable is accounted for. In Tables J5-J8 in S1 Appendix, we added a lagged *mean climate variable* corresponding to the 7-days or 14 days lags (depending on the specification considered) of the mean of each climate variable computed on a 30-days rolling-window. Again, the baseline effects of temperature, solar radiation and humidity remain robust.

### Alternative dataset and longer time span (until 15th September 2020)

We used alternative data to check the robustness of the effects derived with our benchmark dataset from UK met. To this aim, we used data from Dark Sky (https://darksky.net/poweredby/) between 1st January and 15th September. These climatic data were collected in a more general project about Covid-19 data by [37]. We used mean temperatures (in degrees Celsius), relative humidity (percent out of 100), UV index (1 to 7), the probability of precipitation occurring (out of 100), and the wind speed in meters per second. The correlation between the two datasets was checked and was comprised between 0.77 and 0.91 except for the humidity index.

We showed in Tables F5-F8 in S1 Appendix that our results are robust to the use of alternative climatic indicators. In addition, our results are robust to changes in the sample. It was particularly interesting that qualitatively, the results were similar to our baseline results. Temperature and solar radiation were the most influential drivers of Covid-19 outcomes, both in terms of direct and indirect effects, whereas precipitation and wind speed did not display robust significant effects. It is however difficult to conclude about the relative humidity index (not significant at 5% level with the second dataset) considering the lack of matching between the two datasets. Note that our baseline results are also robust when we dropped Chile from our sample, since it represents an outlier country in terms of dynamic associated with the Covid-19 epidemic (see Tables G1-G4 in the S1 Appendix).

## Discussion and conclusion

Understanding links between climate and Covid-19 is crucial. If climatic factors have a physical impact on the virus, it would be helpful and a supplementary weapon to stop the virus in complement to sanitary policies implemented by the authorities.

Our results revealed that the choice of delay and time perspective of the effects of climatic factors on the virus, as well as the choice of Covid-19 outcomes, are crucial and can explain the disparate findings reported in previous literature. Concerning the direct effects of climate on Covid-19, our work highlighted that temperature, humidity and, more interestingly, solar radiation, which has been under considered in previous studies, are significant climatic drivers of the Covid-19 epidemic, although they are associated with a relatively small estimated magnitude. Thus, climatic factors (especially temperature and solar radiation) had a negative direct coefficient, meaning that they are associated with a significant decrease in the number of infected cases and in the fatality rate. Other climatic factors, namely precipitation and wind speed seemed less prone to impact Covid-19 outcomes.

Our estimates pointed out that the way climate factors influence the dynamic of the Covid-19 epidemic is complex to assess due to several econometric issues and, above all, because of potential indirect effects through human behaviour that should be considered simultaneously. Indeed, interrelationships between some climatic variables and mobility were significantly positive. The net effect from climate on Covid-19 outbreaks will, thus, result from some potential direct negative effects of climatic variables and from indirect effects through changes in human behaviour in relation to climatic conditions. Our results showed that direct negative effects from climatic (temperature, humidity and solar radiation) factors on Covid-19 outcomes, when they are significant, are likely to be slightly compensated for by positive indirect effects through human behaviour and mobility.

This does not mean that climate had a neglected role in Covid-19 epidemics. This simply means that climate had a marginal direct effect, significant, *via* physical channels, but it can be compensated for by human behaviour. Indeed, climate can influence other crucial determinants, such as social distancing, wearing masks through mobility, and human behaviour. In this perspective, meteorological factors are drivers of the mobility of many people. On the one hand, high temperatures and sunshine lead people to move away and not stay in clustering places that reduce the transmission rate by introducing more distance between people since it is now well known that the virus is essentially transmitted through droplets generated *via* coughing and sneezing and to a lesser extent through the air, when tiny particles, or aerosols, hang around. A minimum one-meter distance is requested, but a four-meter distance has also been suggested very recently [38]. Climatic factors can, thus, reduce the spread of the virus through this indirect channel. On the other hand, when climate conditions are favourable and people are spending time outdoors, they can go to parks, in meetings, and on terraces; this can sometimes lead to careless behaviour when people reduce social distancing or do not wear masks when it is necessary. In addition, when temperatures are very high, people are likely to stay indoors with air conditioning, leading to an increase in the Covid-19 transmission rate.

### Limitations and further works

In this paper, we performed a robust panel dynamic econometric study with aggregated data on a panel of 37 OECD countries over the January-July (and then September) 2020 period. By definition, it does not cover all countries from Northern and Southern Hemispheres and hence very different climatic regimes. Although we applied some adjustments, focused on relative homogeneous countries and conducted some robustness checks, all countries did not start the epidemic wave exactly at the same time. In the opposite side, all countries have been also influenced by internal factors (demographics, health infrastructures, travels connexions with China, etc.): although these factors are considered through the lags and the fixed effects added in our econometric model, it is not possible to completely take into account them.

As a consequence, increasing further the cross-country heterogeneity, adding the second wave of the Covid-19 epidemic period or including more control variables (health expenditures, population over 65 age) are potential extensions when more data will be available in the future. On an econometric point of view, a better fitting of the distribution of Covid-19 variables using fractional Logit or Probit or non-linear Panel data models (such as Panel Threshold Regression, PTR, models, for example) might be an interesting robustness check.

### What are the implications for policy?

Finally, warmer seasons could only slightly enhance the effect of stringent social distancing. Thus, high temperatures were not a substitute for suitable hygienic and social distancing measures. Consequently, suitable hygienic and public policies should consist of mandating mask wearing and temporarily reducing individual mobility.

### What are the implications of this work for the future?

This work could be extended by introducing air quality and pollution factors as potential supplementary drivers in our model since it is likely that polluting activities can increase the virus intensity. More generally, investigating relationships between climate, meteorological factors, and viruses is important to understand how climate change might influence epidemic dynamics and outbreaks in the coming years.

Moreover, a UNO report [39] entitled "Preventing the next pandemic" concluded that zoonotic diseases are likely to increase in the near future. Climate change and unsustainable human activities are drivers of the increasing frequency of pathogenic microorganisms jumping from animals to people. Beyond the link between climate and the Covid-19 pandemic, it is necessary to better investigate the links between climate and human behaviour and the link between climate and moving borders between humans and animals that are in the epicentre of disease transmission.

## Supporting information

S1 Appendix (DOCX)

S1 Data (XLSX)

S1 File (DO)
