# Supplementary material for: Meteorological factors against COVID-19 and the role of human mobility
Source: PLoS One. 2021 Jun 4;16(6):e0252405. doi: 10.1371/journal.pone.0252405 (PMC8177552; doi:10.1371/journal.pone.0252405)
Supplement: S1 Appendix — (DOCX) [file pone.0252405.s001.docx]

**Meteorological factors against COVID-19 and the role of human mobility**

**Olivier Damette, Clément Mathonnat, and Stéphane Goutte**

**Online Appendix**

**A. Sample**

Australia, Austria, Belgium, Canada, Chile, Colombia, Czechia, Denmark, Estonia, Finland, France, Germany, Greece, Hungary, Ireland, Iceland, Israel, Italy, Japan, Lithuania, Luxembourg, Latvia, Mexico, Netherlands, Norway, New Zealand, Poland, Portugal, Slovakia, Slovenia, Spain, South Korea, Sweden, Switzerland, Turkey, United Kingdom, United States

For the Covid-19 infected cases variable (*casepop*): estimates start on 01/02/2020 for all countries in our sample.

For the Covid-19 fatalities variable (*deathpop*): estimates start on 15/02/2020 for all countries in our sample.

**B. Testing for thresholds effects in climate variables**

**B1. Direct effects of climate variables on Covid-19 infected cases**

|  | **Covid-19 infected cases** | | | | | | | | | |
| --- | --- | --- | --- | --- | --- | --- | --- | --- | --- | --- |
|  | DFE | MG | DFE | MG | DFE | MG | DFE | MG | DFE | MG |
|  | (1) | (2) | (3) | (4) | (5) | (6) | (7) | (8) | (9) | (10) |
| casepop (t-1) | 0.698*** | 0.612*** | 0.698*** | 0.600*** | 0.698*** | 0.608*** | 0.702*** | 0.628*** | 0.702*** | 0.628*** |
|  | [0.0358] | [0.0340] | [0.0358] | [0.0347] | [0.0362] | [0.0351] | [0.0357] | [0.0332] | [0.0356] | [0.0328] |
| casepop (t-7) | 0.243*** | 0.259*** | 0.245*** | 0.247*** | 0.243*** | 0.253*** | 0.244*** | 0.255*** | 0.244*** | 0.257*** |
|  | [0.0355] | [0.0302] | [0.0360] | [0.0294] | [0.0352] | [0.0303] | [0.0356] | [0.0298] | [0.0355] | [0.0300] |
| temperature (t-7) | -0.113** | -0.908 |  |  |  |  |  |  |  |  |
|  | [0.0468] | [0.722] |  |  |  |  |  |  |  |  |
| temperature² (t-7) | 0.0535* | 0.275 |  |  |  |  |  |  |  |  |
|  | [0.0304] | [0.286] |  |  |  |  |  |  |  |  |
| solar radiation (t-7) |  |  | -0.109** | -0.0368 |  |  |  |  |  |  |
|  |  |  | [0.0426] | [0.0630] |  |  |  |  |  |  |
| solar radiation² (t-7) |  |  | -0.0489* | -0.173*** |  |  |  |  |  |  |
|  |  |  | [0.0277] | [0.0323] |  |  |  |  |  |  |
| humidity (t-7) |  |  |  |  | -0.0716* | -0.0170 |  |  |  |  |
|  |  |  |  |  | [0.0357] | [0.0913] |  |  |  |  |
| humidity² (t-7) |  |  |  |  | 0.0788** | 0.0653 |  |  |  |  |
|  |  |  |  |  | [0.0370] | [0.0587] |  |  |  |  |
| precipitation (t-7) |  |  |  |  |  |  | 0.0140 | -0.0275 |  |  |
|  |  |  |  |  |  |  | [0.0278] | [0.0424] |  |  |
| precipitation² (t-7) |  |  |  |  |  |  | -0.00842 | -0.00151 |  |  |
|  |  |  |  |  |  |  | [0.00508] | [0.0117] |  |  |
| wind speed (t-7) |  |  |  |  |  |  |  |  | -0.0232 | -0.0123 |
|  |  |  |  |  |  |  |  |  | [0.0308] | [0.0520] |
| wind speed² (t-7) |  |  |  |  |  |  |  |  | -0.00333 | 0.0867 |
|  |  |  |  |  |  |  |  |  | [0.0195] | [0.0639] |
| Trend | Yes | Yes | Yes | Yes | Yes | Yes | Yes | Yes | Yes | Yes |
| Observations | 6,438 | 6,438 | 6,438 | 6,438 | 6,438 | 6,438 | 6,438 | 6,438 | 6,438 | 6,438 |
| Country | 37 | 37 | 37 | 37 | 37 | 37 | 37 | 37 | 37 | 37 |
| R-squared | 0.821 |  | 0.822 |  | 0.821 |  | 0.821 |  | 0.821 |  |

Note: the coefficients displayed are marginal effects. Standard errors (robust to within-country correlations for DFE) are reported in brackets. *** p < 0.01, ** < 0.05, * p < 0.1. For example, we can see that temperature (t-7) in the first column (DFE estimates) is negative at 5% significance level and temperature² (t-7) is positive and significant at 10% level. As a consequence, the increase in temperatures leads to a reduction in the number of infected cases (with a 7 days lag), but when the temperatures are relatively too high and surpass a threshold, this effect turns negative (effect associated with the positive quadratic term).

**B2. Direct effects of climate variables on Covid-19 fatalities**

|  | **Covid-19 fatalities** | | | | | | | | | |
| --- | --- | --- | --- | --- | --- | --- | --- | --- | --- | --- |
|  | DFE | MG | DFE | MG | DFE | MG | DFE | MG | DFE | MG |
|  | (1) | (2) | (3) | (4) | (5) | (6) | (7) | (8) | (9) | (10) |
| deathpop (t-1) | 0.588*** | 0.355*** | 0.586*** | 0.341*** | 0.589*** | 0.350*** | 0.593*** | 0.373*** | 0.592*** | 0.367*** |
|  | [0.112] | [0.0482] | [0.112] | [0.0469] | [0.111] | [0.0483] | [0.111] | [0.0477] | [0.111] | [0.0491] |
| deathpop (t-14) | 0.152*** | 0.0970*** | 0.151*** | 0.0920*** | 0.154*** | 0.100*** | 0.153*** | 0.103*** | 0.152*** | 0.102*** |
|  | [0.0466] | [0.0254] | [0.0470] | [0.0259] | [0.0464] | [0.0258] | [0.0467] | [0.0253] | [0.0461] | [0.0253] |
| casepop (t-14) | 0.00825** | 0.0189*** | 0.00851** | 0.0179*** | 0.00803** | 0.0185*** | 0.00865** | 0.0185*** | 0.00877** | 0.0189*** |
|  | [0.00334] | [0.00407] | [0.00342] | [0.00412] | [0.00340] | [0.00405] | [0.00345] | [0.00418] | [0.00346] | [0.00418] |
| temperature (t-14) | -0.0171*** | -0.0366** |  |  |  |  |  |  |  |  |
|  | [0.00521] | [0.0163] |  |  |  |  |  |  |  |  |
| temperature² (t-14) | -0.00563 | 0.00406 |  |  |  |  |  |  |  |  |
|  | [0.00439] | [0.0103] |  |  |  |  |  |  |  |  |
| solar radiation (t-14) |  |  | -0.00148 | 0.0170** |  |  |  |  |  |  |
|  |  |  | [0.00411] | [0.00824] |  |  |  |  |  |  |
| solar radiation² (t-14) |  |  | -0.0137*** | -0.0175*** |  |  |  |  |  |  |
|  |  |  | [0.00474] | [0.00642] |  |  |  |  |  |  |
| humidity (t-14) |  |  |  |  | -0.0181*** | -0.00966* |  |  |  |  |
|  |  |  |  |  | [0.00604] | [0.00558] |  |  |  |  |
| humidity² (t-14) |  |  |  |  | 0.00495 | -0.00575 |  |  |  |  |
|  |  |  |  |  | [0.00384] | [0.00625] |  |  |  |  |
| precipitation (t-14) |  |  |  |  |  |  | -0.00453 | -0.00873* |  |  |
|  |  |  |  |  |  |  | [0.00325] | [0.00531] |  |  |
| precipitation² (t-14) |  |  |  |  |  |  | 0.00126 | 0.00260 |  |  |
|  |  |  |  |  |  |  | [0.000938] | [0.00249] |  |  |
| wind speed (t-14) |  |  |  |  |  |  |  |  | 0.00127 | 0.00698 |
|  |  |  |  |  |  |  |  |  | [0.00339] | [0.0142] |
| wind speed² (t-14) |  |  |  |  |  |  |  |  | -0.00279* | 0.00205 |
|  |  |  |  |  |  |  |  |  | [0.00139] | [0.0150] |
| Trend | Yes | Yes | Yes | Yes | Yes | Yes | Yes | Yes | Yes | Yes |
| Observations | 5,920 | 5,920 | 5,920 | 5,920 | 5,920 | 5,920 | 5,920 | 5,920 | 5,920 | 5,920 |
| Country | 37 | 37 | 37 | 37 | 37 | 37 | 37 | 37 | 37 | 37 |
| R-squared | 0.564 |  | 0.564 |  | 0.563 |  | 0.562 |  | 0.563 |  |

Note: the coefficients displayed are marginal effects. Standard errors (robust to within-country correlations for DFE) are reported in brackets. *** p < 0.01, ** < 0.05, * p < 0.1. For example, we can see that temperature (t-14) in the first column (DFE estimates) is negative at 1% significance level and temperature² (t-14) is not significant. As a consequence, the increase in temperatures leads to a reduction in the fatality rate (with a 14 days lag) with no threshold effect.

**C. Testing for interactions between climate variables**

**Table C1. Direct effects of climate variables on Covid-19 infected cases**

|  | **Covid-19 infected cases** | | | | | |
| --- | --- | --- | --- | --- | --- | --- |
|  | DFE | MG | DFE | MG | DFE | MG |
|  | (1) | (2) | (3) | (4) | (5) | (6) |
| casepop (t-1) | 0.698*** | 0.599*** | 0.696*** | 0.602*** | 0.697*** | 0.605*** |
|  | [0.0358] | [0.0349] | [0.0362] | [0.0349] | [0.0358] | [0.0346] |
| casepop (t-7) | 0.245*** | 0.251*** | 0.244*** | 0.259*** | 0.246*** | 0.252*** |
|  | [0.0361] | [0.0299] | [0.0357] | [0.0304] | [0.0362] | [0.0300] |
| temperature (t-7) | -0.0574 | -0.170** | -0.227*** | -0.301*** |  |  |
|  | [0.0460] | [0.0845] | [0.0822] | [0.108] |  |  |
| solar radiation (t-7) | -0.0918** | -0.0669 |  |  | -0.105** | -0.261 |
|  | [0.0428] | [0.0766] |  |  | [0.0421] | [0.160] |
| humidity (t-7) |  |  | 0.119* | 0.0815 | -0.0421 | -0.186** |
|  |  |  | [0.0625] | [0.0875] | [0.0328] | [0.0893] |
| temperature*solar radiation (t-7) | 0.0294 | -0.104* |  |  |  |  |
|  | [0.0321] | [0.0610] |  |  |  |  |
| temperature*humidity (t-7) |  |  | 0.0590 | 0.00972 |  |  |
|  |  |  | [0.0401] | [0.0661] |  |  |
| solar radiation*humidity (t-7) |  |  |  |  | 0.0728* | 0.0384 |
|  |  |  |  |  | [0.0360] | [0.0721] |
| Trend | Yes | Yes | Yes | Yes | Yes | Yes |
| Observations | 6,438 | 6,438 | 6,438 | 6,438 | 6,438 | 6,438 |
| Country | 0.822 |  | 0.822 |  | 0.822 |  |
| R-squared | 37 | 37 | 37 | 37 | 37 | 37 |

Note: the coefficients displayed are marginal effects. Standard errors (robust to within-country correlations for DFE) are reported in brackets. *** p < 0.01, ** < 0.05, * p < 0.1.

**Table C2. Direct effects of climate variables on Covid-19 fatalities**

|  | **Covid-19 fatalities** | | | | | |
| --- | --- | --- | --- | --- | --- | --- |
|  | DFE | MG | DFE | MG | DFE | MG |
|  | (1) | (2) | (3) | (4) | (5) | (6) |
| deathpop (t-1) | 0.585*** | 0.338*** | 0.589*** | 0.348*** | 0.589*** | 0.344*** |
|  | [0.113] | [0.0478] | [0.112] | [0.0482] | [0.112] | [0.0485] |
| deathpop (t-14) | 0.150*** | 0.0879*** | 0.154*** | 0.0987*** | 0.151*** | 0.0884*** |
|  | [0.0464] | [0.0259] | [0.0469] | [0.0257] | [0.0468] | [0.0258] |
| casepop (t-14) | 0.00825** | 0.0179*** | 0.00814** | 0.0188*** | 0.00840** | 0.0183*** |
|  | [0.00332] | [0.00407] | [0.00340] | [0.00412] | [0.00336] | [0.00412] |
| temperature (t-14) | -0.0148*** | -0.0145* | -0.0153* | 0.00282 |  |  |
|  | [0.00530] | [0.00756] | [0.00783] | [0.0120] |  |  |
| solar radiation (t-14) | 0.00245 | 0.00482 |  |  | -0.00302 | 0.00266 |
|  | [0.00401] | [0.00945] |  |  | [0.00358] | [0.00719] |
| humidity (t-14) |  |  | -0.00253 | -0.0108 | -0.0104** | -0.0145*** |
|  |  |  | [0.00752] | [0.00891] | [0.00415] | [0.00561] |
| temperature*solar radiation (t-14) | -0.0109** | -0.00629 |  |  |  |  |
|  | [0.00497] | [0.00828] |  |  |  |  |
| temperature*humidity (t-14) |  |  | -0.00131 | -0.00425 |  |  |
|  |  |  | [0.00517] | [0.00890] |  |  |
| solar radiation*humidity (t-14) |  |  |  |  | -0.00646 | -0.00516 |
|  |  |  |  |  | [0.00455] | [0.00738] |
| Trend | Yes | Yes | Yes | Yes | Yes | Yes |
| Observations | 5,920 | 5,920 | 5,920 | 5,920 | 5,920 | 5,920 |
| Country | 37 | 37 | 37 | 37 | 37 | 37 |
| R-squared | 0.564 |  | 0.563 |  | 0.563 |  |

Note: the coefficients displayed are marginal effects. Standard errors (robust to within-country correlations for DFE) are reported in brackets. *** p < 0.01, ** < 0.05, * p < 0.1.

**D. Increasing lags for Covid-19, climate, and human mobility variables**

**Table D1. Direct effects of climate variables on Covid-19 infected cases (lag 21)**

|  | **Covid-19 infected cases** | | | | | | | | | |
| --- | --- | --- | --- | --- | --- | --- | --- | --- | --- | --- |
|  | DFE | MG | DFE | MG | DFE | MG | DFE | MG | DFE | MG |
|  | (1) | (2) | (3) | (4) | (5) | (6) | (7) | (8) | (9) | (10) |
| casepop (t-1) | 0.880*** | 0.755*** | 0.879*** | 0.760*** | 0.883*** | 0.753*** | 0.883*** | 0.763*** | 0.883*** | 0.764*** |
|  | [0.0185] | [0.0337] | [0.0185] | [0.0329] | [0.0185] | [0.0334] | [0.0188] | [0.0331] | [0.0188] | [0.0329] |
| casepop (t-21) | 0.0316*** | 0.0920* | 0.0361*** | 0.0985** | 0.0346*** | 0.0928** | 0.0356*** | 0.0965** | 0.0357*** | 0.0976** |
|  | [0.00781] | [0.0475] | [0.00787] | [0.0473] | [0.00768] | [0.0473] | [0.00734] | [0.0465] | [0.00739] | [0.0467] |
| temperature (t-21) | -0.130** | -0.201*** |  |  |  |  |  |  |  |  |
|  | [0.0638] | [0.0631] |  |  |  |  |  |  |  |  |
| solar radiation (t-21) |  |  | -0.133** | -0.129* |  |  |  |  |  |  |
|  |  |  | [0.0555] | [0.0665] |  |  |  |  |  |  |
| humidity (t-21) |  |  |  |  | -0.0254 | -0.104 |  |  |  |  |
|  |  |  |  |  | [0.0503] | [0.0789] |  |  |  |  |
| precipitation (t-21) |  |  |  |  |  |  | 0.00892 | -0.00307 |  |  |
|  |  |  |  |  |  |  | [0.0214] | [0.0262] |  |  |
| wind speed (t-21) |  |  |  |  |  |  |  |  | 0.0115 | -0.0616 |
|  |  |  |  |  |  |  |  |  | [0.0184] | [0.0715] |
| Trend | Yes | Yes | Yes | Yes | Yes | Yes | Yes | Yes | Yes | Yes |
| Observations | 6,438 | 6,438 | 6,438 | 6,438 | 6,438 | 6,438 | 6,438 | 6,438 | 6,438 | 6,438 |
| Country | 37 | 37 | 37 | 37 | 37 | 37 | 37 | 37 | 37 | 37 |
| R-squared | 0.806 |  | 0.806 |  | 0.805 |  | 0.805 |  | 0.805 |  |

Note: the coefficients displayed are marginal effects. Standard errors (robust to within-country correlations for DFE) are reported in brackets. *** p < 0.01, ** < 0.05, * p < 0.1.

**Table D2. Direct effects of climate variables on Covid-19 fatalities (lag 28)**

|  | **Covid-19 fatalities** | | | | | | | | | |  |  |
| --- | --- | --- | --- | --- | --- | --- | --- | --- | --- | --- | --- | --- |
|  | DFE | MG | DFE | MG | DFE | MG | DFE | MG | DFE | MG |  |  |
|  | (1) | (2) | (3) | (4) | (5) | (6) | (7) | (8) | (9) | (10) |  |  |
| deathpop (t-1) | 0.702*** | 0.519*** | 0.705*** | 0.524*** | 0.704*** | 0.515*** | 0.707*** | 0.526*** | 0.707*** | 0.529*** |  |  |
|  | [0.0971] | [0.0472] | [0.0963] | [0.0466] | [0.0965] | [0.0468] | [0.0950] | [0.0469] | [0.0951] | [0.0468] |  |  |
| deathpop (t-28) | 0.00247 | -0.00958 | 0.00502 | -0.00868 | -0.000746 | -0.0151 | 0.00114 | -0.0110 | 0.00280 | -0.00773 |  |  |
|  | [0.0251] | [0.0208] | [0.0259] | [0.0204] | [0.0247] | [0.0207] | [0.0254] | [0.0206] | [0.0256] | [0.0208] |  |  |
| casepop (t-28) | 0.00496 | 0.00817*** | 0.00544 | 0.00840*** | 0.00523 | 0.00757*** | 0.00568 | 0.00822*** | 0.00565 | 0.00847*** |  |  |
|  | [0.00326] | [0.00291] | [0.00335] | [0.00287] | [0.00348] | [0.00279] | [0.00367] | [0.00283] | [0.00369] | [0.00284] |  |  |
| temperature (t-28) | -0.0198** | -0.0147 |  |  |  |  |  |  |  |  |  |  |
|  | [0.00859] | [0.00994] |  |  |  |  |  |  |  |  |  |  |
| solar radiation (t-28) |  |  | -0.0104* | 0.000218 |  |  |  |  |  |  |  |  |
|  |  |  | [0.00575] | [0.00490] |  |  |  |  |  |  |  |  |
| humidity (t-28) |  |  |  |  | -0.0144** | -0.0224*** |  |  |  |  |  |  |
|  |  |  |  |  | [0.00692] | [0.00813] |  |  |  |  |  |  |
| precipitation (t-28) |  |  |  |  |  |  | 0.00220 | -0.00354 |  |  |  |  |
|  |  |  |  |  |  |  | [0.00256] | [0.00279] |  |  |  |  |
| wind speed (t-28) |  |  |  |  |  |  |  |  | 0.00624** | 0.00541* |  |  |
|  |  |  |  |  |  |  |  |  | [0.00283] | [0.00286] |  |  |
| Trend | Yes | Yes | Yes | Yes | Yes | Yes | Yes | Yes | Yes | Yes |  |  |
| Observations | 5,920 | 5,920 | 5,920 | 5,920 | 5,920 | 5,920 | 5,920 | 5,920 | 5,920 | 5,920 |  |  |
| Country | 37 | 37 | 37 | 37 | 37 | 37 | 37 | 37 | 37 | 37 |  |  |
| R-squared | 0.533 |  | 0.533 |  | 0.533 |  | 0.532 |  | 0.533 |  |  |  |

Note: the coefficients displayed are marginal effects. Standard errors (robust to within-country correlations for DFE) are reported in brackets. *** p < 0.01, ** < 0.05, * p < 0.1.

**Table D3. Indirect effects of climate variables on Covid-19 infected cases through human mobility (lag 21)**

|  | **Covid-19 infected cases** | | | | | | | | | |
| --- | --- | --- | --- | --- | --- | --- | --- | --- | --- | --- |
|  | DFE | MG | DFE | MG | DFE | MG | DFE | MG | DFE | MG |
|  | (1) | (2) | (3) | (4) | (5) | (6) | (7) | (8) | (9) | (10) |
| casepop (t-1) | 0.877*** | 0.703*** | 0.876*** | 0.710*** | 0.880*** | 0.709*** | 0.881*** | 0.725*** | 0.880*** | 0.729*** |
|  | [0.0177] | [0.0363] | [0.0177] | [0.0349] | [0.0175] | [0.0362] | [0.0177] | [0.0349] | [0.0177] | [0.0343] |
| casepop (t-21) | 0.0444*** | 0.101** | 0.0434*** | 0.107** | 0.0463*** | 0.0986** | 0.0444*** | 0.102** | 0.0456*** | 0.101** |
|  | [0.00825] | [0.0487] | [0.00794] | [0.0480] | [0.00771] | [0.0485] | [0.00718] | [0.0481] | [0.00723] | [0.0474] |
| mobility (t-21) | 0.00292*** | 0.00116 | 0.00159 | 0.000221 | 0.00262*** | 0.000187 | 0.00193** | 0.00138 | 0.00213** | 0.00199 |
|  | [0.000927] | [0.00158] | [0.00103] | [0.00123] | [0.000810] | [0.00136] | [0.000792] | [0.00154] | [0.000864] | [0.00168] |
| temperature (t-21) | -0.0990 | -0.467** |  |  |  |  |  |  |  |  |
|  | [0.109] | [0.217] |  |  |  |  |  |  |  |  |
| temperature*mobility (t-21) | -0.000809 | 0.00291 |  |  |  |  |  |  |  |  |
|  | [0.00120] | [0.00250] |  |  |  |  |  |  |  |  |
| solar radiation (t-21) |  |  | -0.175 | -0.489** |  |  |  |  |  |  |
|  |  |  | [0.146] | [0.199] |  |  |  |  |  |  |
| solar radiation*mobility (t-21) |  |  | 0.000475 | 0.00508** |  |  |  |  |  |  |
|  |  |  | [0.00141] | [0.00221] |  |  |  |  |  |  |
| humidity (t-21) |  |  |  |  | 0.0166 | -0.346* |  |  |  |  |
|  |  |  |  |  | [0.0919] | [0.183] |  |  |  |  |
| humidity*mobility (t-21) |  |  |  |  | -0.000979 | 0.00290 |  |  |  |  |
|  |  |  |  |  | [0.000904] | [0.00238] |  |  |  |  |
| precipitation (t-21) |  |  |  |  |  |  | -0.00659 | 0.0755 |  |  |
|  |  |  |  |  |  |  | [0.0772] | [0.107] |  |  |
| precipitation*mobility (t-21) |  |  |  |  |  |  | 0.000152 | -0.000421 |  |  |
|  |  |  |  |  |  |  | [0.000697] | [0.00109] |  |  |
| wind speed (t-21) |  |  |  |  |  |  |  |  | -0.121 | -0.173 |
|  |  |  |  |  |  |  |  |  | [0.117] | [0.144] |
| wind speed*mobility (t-21) |  |  |  |  |  |  |  |  | 0.00143 | 0.00154 |
|  |  |  |  |  |  |  |  |  | [0.00110] | [0.00122] |
| Trend | Yes | Yes | Yes | Yes | Yes | Yes | Yes | Yes | Yes | Yes |
| Observations | 6,290 | 6,290 | 6,290 | 6,290 | 6,290 | 6,290 | 6,290 | 6,290 | 6,290 | 6,290 |
| Country | 37 | 37 | 37 | 37 | 37 | 37 | 37 | 37 | 37 | 37 |
| R-squared | 0.804 |  | 0.804 |  | 0.804 |  | 0.803 |  | 0.804 |  |

Note: the coefficients displayed are marginal effects. Standard errors (robust to within-country correlations for DFE) are reported in brackets. *** p < 0.01, ** < 0.05, * p < 0.1.

**Table D4. Indirect effects of climate variables on Covid-19 fatalities through human mobility**

|  | **Covid-19 fatalities** | | | | | | | | | |
| --- | --- | --- | --- | --- | --- | --- | --- | --- | --- | --- |
|  | DFE | MG | DFE | MG | DFE | MG | DFE | MG | DFE | MG |
|  | (1) | (2) | (3) | (4) | (5) | (6) | (7) | (8) | (9) | (10) |
| deathpop (t-1) | 0.712*** | 0.447*** | 0.712*** | 0.456*** | 0.715*** | 0.448*** | 0.718*** | 0.469*** | 0.717*** | 0.468*** |
|  | [0.0893] | [0.0495] | [0.0903] | [0.0484] | [0.0885] | [0.0492] | [0.0875] | [0.0480] | [0.0876] | [0.0483] |
| deathpop (t-28) | 0.00259 | -0.0183 | 0.00336 | -0.0198 | -0.00270 | -0.0220 | -0.00398 | -0.0351 | -0.00163 | -0.0332 |
|  | [0.0239] | [0.0229] | [0.0247] | [0.0219] | [0.0235] | [0.0226] | [0.0236] | [0.0225] | [0.0240] | [0.0232] |
| casepop (t-28) | 0.00352 | 0.00428 | 0.00372 | 0.00454 | 0.00405 | 0.00438 | 0.00460 | 0.00440 | 0.00454 | 0.00510* |
|  | [0.00302] | [0.00313] | [0.00286] | [0.00296] | [0.00328] | [0.00320] | [0.00329] | [0.00300] | [0.00338] | [0.00302] |
| mobility (t-28) | -0.000181* | -0.000307 | -0.000345** | -0.000541*** | -0.000165* | -0.000415** | -0.000229** | -0.000597** | -0.000226** | -0.000563*** |
|  | [9.78e-05] | [0.000200] | [0.000140] | [0.000183] | [9.07e-05] | [0.000172] | [9.54e-05] | [0.000237] | [0.000106] | [0.000218] |
| temperature (t-28) | -0.0410** | -0.0454 |  |  |  |  |  |  |  |  |
|  | [0.0170] | [0.0388] |  |  |  |  |  |  |  |  |
| temperature*mobility (t-28) | 0.000238* | 0.000391 |  |  |  |  |  |  |  |  |
|  | [0.000137] | [0.000381] |  |  |  |  |  |  |  |  |
| solar radiation (t-28) |  |  | -0.0428** | -0.0379*** |  |  |  |  |  |  |
|  |  |  | [0.0185] | [0.0147] |  |  |  |  |  |  |
| solar radiation*mobility (t-28) |  |  | 0.000335** | 0.000460** |  |  |  |  |  |  |
|  |  |  | [0.000162] | [0.000221] |  |  |  |  |  |  |
| humidity (t-28) |  |  |  |  | -0.0275* | -0.0516 |  |  |  |  |
|  |  |  |  |  | [0.0147] | [0.0355] |  |  |  |  |
| humidity*mobility (t-28) |  |  |  |  | 0.000167 | 0.000415 |  |  |  |  |
|  |  |  |  |  | [0.000120] | [0.000377] |  |  |  |  |
| precipitation (t-28) |  |  |  |  |  |  | 0.00662 | -0.0123 |  |  |
|  |  |  |  |  |  |  | [0.00561] | [0.00997] |  |  |
| precipitation*mobility (t-28) |  |  |  |  |  |  | -4.42e-05 | 0.000161 |  |  |
|  |  |  |  |  |  |  | [5.83e-05] | [0.000157] |  |  |
| wind speed (t-28) |  |  |  |  |  |  |  |  | 0.0133 | 0.00470 |
|  |  |  |  |  |  |  |  |  | [0.0105] | [0.0119] |
| wind speed*mobility (t-28) |  |  |  |  |  |  |  |  | -8.21e-05 | 3.11e-05 |
|  |  |  |  |  |  |  |  |  | [9.83e-05] | [0.000123] |
| Trend | Yes | Yes | Yes | Yes | Yes | Yes | Yes | Yes | Yes | Yes |
| Observations | 5,846 | 5,846 | 5,846 | 5,846 | 5,846 | 5,846 | 5,846 | 5,846 | 5,846 | 5,846 |
| Country | 37 | 37 | 37 | 37 | 37 | 37 | 37 | 37 | 37 | 37 |
| R-squared | 0.553 |  | 0.553 |  | 0.553 |  | 0.552 |  | 0.552 |  |

Note: the coefficients displayed are marginal effects. Standard errors (robust to within-country correlations for DFE) are reported in brackets. *** p < 0.01, ** < 0.05, * p < 0.1.

**E. Endogeneity issues: System-GMM estimates**

**Table E1. Indirect effects of climate variables on Covid-19 infected cases through human mobility**

|  | **Covid-19 infected cases** | | | | |
| --- | --- | --- | --- | --- | --- |
|  | (1) | (2) | (3) | (4) | (5) |
| casepop (t-1) | 0.663*** | 0.628*** | 0.673*** | 0.676*** | 0.659*** |
|  | [0.0347] | [0.0457] | [0.0324] | [0.0374] | [0.0378] |
| casepop (t-7) | 0.235*** | 0.267*** | 0.241*** | 0.240*** | 0.231*** |
|  | [0.0445] | [0.0509] | [0.0421] | [0.0468] | [0.0439] |
| mobility (t-7) | -0.00717** | -0.00549 | -0.00605** | -0.00312 | -0.0138** |
|  | [0.00307] | [0.00443] | [0.00297] | [0.00470] | [0.00694] |
| temperature (t-7) | -1.722*** |  |  |  |  |
|  | [0.651] |  |  |  |  |
| temperature*mobility (t-7) | 0.0183*** |  |  |  |  |
|  | [0.00658] |  |  |  |  |
| solar radiation (t-7) |  | -4.024*** |  |  |  |
|  |  | [1.310] |  |  |  |
| solar radiation*mobility (t-7) |  | 0.0410*** |  |  |  |
|  |  | [0.0136] |  |  |  |
| humidity (t-7) |  |  | -0.881** |  |  |
|  |  |  | [0.417] |  |  |
| humidity*mobility (t-7) |  |  | 0.00997** |  |  |
|  |  |  | [0.00436] |  |  |
| precipitation (t-7) |  |  |  | -2.231* |  |
|  |  |  |  | [1.174] |  |
| precipitation*mobility (t-7) |  |  |  | 0.0228* |  |
|  |  |  |  | [0.0125] |  |
| wind speed (t-7) |  |  |  |  | 2.475** |
|  |  |  |  |  | [1.021] |
| wind speed*mobility (t-7) |  |  |  |  | -0.0317*** |
|  |  |  |  |  | [0.0117] |
| Trend | Yes | Yes | Yes | Yes | Yes |
| AR(1) p-value | 0.00 | 0.00 | 0.00 | 0.00 | 0.00 |
| AR(2) p-value | 0.93 | 0.82 | 0.90 | 0.95 | 0.43 |
| Hansen test p-value | 0.33 | 0.28 | 0.28 | 0.23 | 0.22 |
| Observations | 6,364 | 6,364 | 6,364 | 6,364 | 6,364 |
| Country | 37 | 37 | 37 | 37 | 37 |

Note: the coefficients displayed are marginal effects. Robust standard errors are reported in brackets. *** p < 0.01, ** < 0.05, * p < 0.1.

**Table E2. Indirect effects of climate variables on Covid-19 fatalities through human mobility**

|  | **Covid-19 fatalities** | | | | |
| --- | --- | --- | --- | --- | --- |
|  | (1) | (2) | (3) | (4) | (5) |
| deathpop (t-1) | 0.257*** | 0.248*** | 0.262*** | 0.272*** | 0.261*** |
|  | [0.0916] | [0.0912] | [0.0928] | [0.0948] | [0.101] |
| deathpop (t-14) | -0.000481 | 0.0321 | -0.0340 | -0.0621 | -0.0365 |
|  | [0.133] | [0.125] | [0.132] | [0.145] | [0.135] |
| casepop (t-14) | 0.0438*** | 0.0464*** | 0.0471*** | 0.0542*** | 0.0508*** |
|  | [0.0123] | [0.0134] | [0.0124] | [0.0164] | [0.0127] |
| mobility (t-14) | -0.00108* | -0.000852** | -0.00102** | -0.000753 | -0.000955** |
|  | [0.000554] | [0.000349] | [0.000499] | [0.000506] | [0.000400] |
| temperature (t-14) | -0.144 |  |  |  |  |
|  | [0.0893] |  |  |  |  |
| temperature*mobility (t-14) | 0.00163* |  |  |  |  |
|  | [0.000939] |  |  |  |  |
| solar radiation (t-14) |  | -0.192*** |  |  |  |
|  |  | [0.0742] |  |  |  |
| solar radiation*mobility (t-14) |  | 0.00199*** |  |  |  |
|  |  | [0.000767] |  |  |  |
| humidity (t-14) |  |  | -0.0796 |  |  |
|  |  |  | [0.0660] |  |  |
| humidity*mobility (t-14) |  |  | 0.000881 |  |  |
|  |  |  | [0.000657] |  |  |
| precipitation (t-14) |  |  |  | 0.0534 |  |
|  |  |  |  | [0.0455] |  |
| precipitation*mobility (t-14) |  |  |  | -0.000528 |  |
|  |  |  |  | [0.000458] |  |
| wind speed (t-14) |  |  |  |  | 0.0703 |
|  |  |  |  |  | [0.0909] |
| wind speed*mobility (t-14) |  |  |  |  | -0.000756 |
|  |  |  |  |  | [0.000900] |
| Trend | Yes | Yes | Yes | Yes | Yes |
| AR(1) p-value | 0.03 | 0.02 | 0.03 | 0.02 | 0.03 |
| AR(2) p-value | 0.67 | 0.58 | 0.69 | 0.66 | 0.63 |
| Hansen test p-value | 0.39 | 0.74 | 0.33 | 0.49 | 0.38 |
| Observations | 5,846 | 5,846 | 5,846 | 5,846 | 5,846 |
| Country | 37 | 37 | 37 | 37 | 37 |

Note: the coefficients displayed are marginal effects. Robust standard errors are reported in brackets. *** p < 0.01, ** < 0.05, * p < 0.1.

**F. Time span change**

**Table F1. Direct effects of climate variables on Covid-19 infected cases: estimates since 01/01/2020**

|  | **Covid-19 infected cases** | | | | | | | | | |
| --- | --- | --- | --- | --- | --- | --- | --- | --- | --- | --- |
|  | DFE | MG | DFE | MG | DFE | MG | DFE | MG | DFE | MG |
|  | (1) | (2) | (3) | (4) | (5) | (6) | (7) | (8) | (9) | (10) |
| casepop (t-1) | 0.703*** | 0.624*** | 0.702*** | 0.628*** | 0.705*** | 0.624*** | 0.705*** | 0.636*** | 0.705*** | 0.637*** |
|  | [0.0357] | [0.0341] | [0.0357] | [0.0334] | [0.0355] | [0.0342] | [0.0356] | [0.0330] | [0.0356] | [0.0328] |
| casepop (t-7) | 0.243*** | 0.256*** | 0.246*** | 0.259*** | 0.245*** | 0.253*** | 0.245*** | 0.258*** | 0.245*** | 0.259*** |
|  | [0.0356] | [0.0300] | [0.0358] | [0.0300] | [0.0358] | [0.0297] | [0.0356] | [0.0299] | [0.0355] | [0.0300] |
| temperature (t-7) | -0.0975** | -0.200** |  |  |  |  |  |  |  |  |
|  | [0.0459] | [0.0793] |  |  |  |  |  |  |  |  |
| solar radiation (t-7) |  |  | -0.0951** | -0.105* |  |  |  |  |  |  |
|  |  |  | [0.0392] | [0.0599] |  |  |  |  |  |  |
| humidity (t-7) |  |  |  |  | -0.0154 | -0.0904 |  |  |  |  |
|  |  |  |  |  | [0.0396] | [0.0570] |  |  |  |  |
| precipitation (t-7) |  |  |  |  |  |  | -0.0166 | -0.0296 |  |  |
|  |  |  |  |  |  |  | [0.0115] | [0.0183] |  |  |
| wind speed (t-7) |  |  |  |  |  |  |  |  | -0.0185 | -0.0871 |
|  |  |  |  |  |  |  |  |  | [0.0129] | [0.0535] |
| Trend | Yes | Yes | Yes | Yes | Yes | Yes | Yes | Yes | Yes | Yes |
| Observations | 7,326 | 7,326 | 7,326 | 7,326 | 7,326 | 7,326 | 7,326 | 7,326 | 7,326 | 7,326 |
| Country | 37 | 37 | 37 | 37 | 37 | 37 | 37 | 37 | 37 | 37 |
| R-squared | 0.834 |  | 0.834 |  | 0.833 |  | 0.833 |  | 0.833 |  |

Note: the coefficients displayed are marginal effects. Standard errors (robust to within-country correlations for DFE) are reported in brackets. *** p < 0.01, ** < 0.05, * p < 0.1.

**Table F2.** **Direct effects of climate variables on Covid-19 fatalities: estimates since 01/01/2020**

|  | **Covid-19 fatalities** | | | | | | | | | |
| --- | --- | --- | --- | --- | --- | --- | --- | --- | --- | --- |
|  | DFE | MG | DFE | MG | DFE | MG | DFE | MG | DFE | MG |
|  | (1) | (2) | (3) | (4) | (5) | (6) | (7) | (8) | (9) | (10) |
| deathpop (t-1) | 0.602*** | 0.379*** | 0.606*** | 0.385*** | 0.601*** | 0.372*** | 0.606*** | 0.392*** | 0.606*** | 0.389*** |
|  | [0.110] | [0.0485] | [0.110] | [0.0491] | [0.110] | [0.0490] | [0.109] | [0.0487] | [0.110] | [0.0495] |
| deathpop (t-14) | 0.163*** | 0.109*** | 0.161*** | 0.105*** | 0.159*** | 0.0983*** | 0.161*** | 0.106*** | 0.161*** | 0.108*** |
|  | [0.0480] | [0.0254] | [0.0487] | [0.0254] | [0.0473] | [0.0252] | [0.0479] | [0.0252] | [0.0476] | [0.0251] |
| casepop (t-14) | 0.00770** | 0.0185*** | 0.00827** | 0.0187*** | 0.00791** | 0.0181*** | 0.00830** | 0.0186*** | 0.00829** | 0.0188*** |
|  | [0.00307] | [0.00417] | [0.00323] | [0.00430] | [0.00308] | [0.00411] | [0.00322] | [0.00427] | [0.00322] | [0.00427] |
| temperature (t-14) | -0.0165*** | -0.0206*** |  |  |  |  |  |  |  |  |
|  | [0.00453] | [0.00600] |  |  |  |  |  |  |  |  |
| solar radiation (t-14) |  |  | -0.000808 | 0.00707 |  |  |  |  |  |  |
|  |  |  | [0.00310] | [0.00555] |  |  |  |  |  |  |
| humidity (t-14) |  |  |  |  | -0.0158*** | -0.0218*** |  |  |  |  |
|  |  |  |  |  | [0.00519] | [0.00527] |  |  |  |  |
| precipitation (t-14) |  |  |  |  |  |  | 0.000795 | 0.00161 |  |  |
|  |  |  |  |  |  |  | [0.00228] | [0.00376] |  |  |
| wind speed (t-14) |  |  |  |  |  |  |  |  | 0.000281 | -0.00197 |
|  |  |  |  |  |  |  |  |  | [0.00190] | [0.00750] |
| Trend | Yes | Yes | Yes | Yes | Yes | Yes | Yes | Yes | Yes | Yes |
| Observations | 7,067 | 7,067 | 7,067 | 7,067 | 7,067 | 7,067 | 7,067 | 7,067 | 7,067 | 7,067 |
| Country | 37 | 37 | 37 | 37 | 37 | 37 | 37 | 37 | 37 | 37 |
| R-squared | 0.591 |  | 0.590 |  | 0.591 |  | 0.590 |  | 0.590 |  |

Note: the coefficients displayed are marginal effects. Standard errors (robust to within-country correlations for DFE) are reported in brackets. *** p < 0.01, ** < 0.05, * p < 0.1.

**Table F3. Indirect effects of climate variables on Covid-19 infected cases through human mobility: estimates since 01/01/2020**

|  | **Covid-19 infected cases** | | | | | | | | | |
| --- | --- | --- | --- | --- | --- | --- | --- | --- | --- | --- |
|  | DFE | MG | DFE | MG | DFE | MG | DFE | MG | DFE | MG |
|  | (1) | (2) | (3) | (4) | (5) | (6) | (7) | (8) | (9) | (10) |
| casepop (t-1) | 0.699*** | 0.527*** | 0.696*** | 0.553*** | 0.702*** | 0.538*** | 0.703*** | 0.586*** | 0.703*** | 0.587*** |
|  | [0.0356] | [0.0405] | [0.0360] | [0.0361] | [0.0352] | [0.0411] | [0.0355] | [0.0361] | [0.0352] | [0.0362] |
| casepop (t-7) | 0.241*** | 0.224*** | 0.244*** | 0.244*** | 0.242*** | 0.220*** | 0.244*** | 0.235*** | 0.244*** | 0.239*** |
|  | [0.0360] | [0.0319] | [0.0363] | [0.0311] | [0.0361] | [0.0310] | [0.0361] | [0.0305] | [0.0360] | [0.0302] |
| mobility (t-7) | -0.000430 | -0.00305 | -0.00162** | -0.00531*** | -0.000514 | -0.00501*** | -0.000244 | -0.00333*** | -0.000236 | -0.00289** |
|  | [0.000636] | [0.00321] | [0.000784] | [0.00139] | [0.000503] | [0.00131] | [0.000492] | [0.00125] | [0.000545] | [0.00125] |
| temperature (t-7) | -0.258*** | -0.715*** |  |  |  |  |  |  |  |  |
|  | [0.0932] | [0.212] |  |  |  |  |  |  |  |  |
| temperature*mobility (t-7) | 0.00152 | 0.00576** |  |  |  |  |  |  |  |  |
|  | [0.000944] | [0.00293] |  |  |  |  |  |  |  |  |
| solar radiation (t-7) |  |  | -0.368*** | -0.613*** |  |  |  |  |  |  |
|  |  |  | [0.129] | [0.194] |  |  |  |  |  |  |
| solar radiation*mobility (t-7) |  |  | 0.00262** | 0.00617*** |  |  |  |  |  |  |
|  |  |  | [0.00109] | [0.00195] |  |  |  |  |  |  |
| humidity (t-7) |  |  |  |  | -0.121* | -0.694*** |  |  |  |  |
|  |  |  |  |  | [0.0656] | [0.138] |  |  |  |  |
| humidity*mobility (t-7) |  |  |  |  | 0.00112* | 0.00688*** |  |  |  |  |
|  |  |  |  |  | [0.000643] | [0.00138] |  |  |  |  |
| precipitation (t-7) |  |  |  |  |  |  | -0.0796 | -0.0760 |  |  |
|  |  |  |  |  |  |  | [0.0503] | [0.0510] |  |  |
| precipitation*mobility (t-7) |  |  |  |  |  |  | 0.000658 | 0.000321 |  |  |
|  |  |  |  |  |  |  | [0.000464] | [0.000535] |  |  |
| wind speed (t-7) |  |  |  |  |  |  |  |  | -0.0511 | 0.0153 |
|  |  |  |  |  |  |  |  |  | [0.0790] | [0.130] |
| wind speed*mobility (t-7) |  |  |  |  |  |  |  |  | 0.000306 | -0.000911 |
|  |  |  |  |  |  |  |  |  | [0.000685] | [0.00140] |
| Trend | Yes | Yes | Yes | Yes | Yes | Yes | Yes | Yes | Yes | Yes |
| Observations | 6,808 | 6,808 | 6,808 | 6,808 | 6,808 | 6,808 | 6,808 | 6,808 | 6,808 | 6,808 |
| Country | 37 | 37 | 37 | 37 | 37 | 37 | 37 | 37 | 37 | 37 |
| R-squared | 0.828 |  | 0.829 |  | 0.828 |  | 0.828 |  | 0.828 |  |

Note: the coefficients displayed are marginal effects. Standard errors (robust to within-country correlations for DFE) are reported in brackets. *** p < 0.01, ** < 0.05, * p < 0.1.

**Table F4. Indirect effects of climate variables on Covid-19 fatalities through human mobility: estimates since 01/01/2020**

|  | **Covid-19 fatalities** | | | | | | | | | |
| --- | --- | --- | --- | --- | --- | --- | --- | --- | --- | --- |
|  | DFE | MG | DFE | MG | DFE | MG | DFE | MG | DFE | MG |
|  | (1) | (2) | (3) | (4) | (5) | (6) | (7) | (8) | (9) | (10) |
| deathpop (t-1) | 0.609*** | 0.270*** | 0.611*** | 0.287*** | 0.609*** | 0.268*** | 0.618*** | 0.299*** | 0.617*** | 0.297*** |
|  | [0.119] | [0.0453] | [0.120] | [0.0458] | [0.120] | [0.0460] | [0.119] | [0.0466] | [0.120] | [0.0493] |
| deathpop (t-14) | 0.147*** | 0.0822*** | 0.145*** | 0.0676** | 0.141*** | 0.0736*** | 0.135** | 0.0564** | 0.136** | 0.0564** |
|  | [0.0510] | [0.0264] | [0.0519] | [0.0269] | [0.0503] | [0.0266] | [0.0506] | [0.0270] | [0.0504] | [0.0269] |
| casepop (t-14) | 0.00511* | 0.0120*** | 0.00573** | 0.0144*** | 0.00559* | 0.0116*** | 0.00674** | 0.0146*** | 0.00669** | 0.0149*** |
|  | [0.00278] | [0.00372] | [0.00265] | [0.00420] | [0.00285] | [0.00368] | [0.00287] | [0.00414] | [0.00292] | [0.00428] |
| mobility (t-14) | -0.000550** | -0.00107*** | -0.000671*** | -0.00113*** | -0.000505*** | -0.00101*** | -0.000469*** | -0.000835*** | -0.000481*** | -0.000820*** |
|  | [0.000203] | [0.000273] | [0.000236] | [0.000248] | [0.000181] | [0.000211] | [0.000169] | [0.000306] | [0.000171] | [0.000288] |
| temperature (t-14) | -0.0579*** | -0.0811*** |  |  |  |  |  |  |  |  |
|  | [0.0184] | [0.0239] |  |  |  |  |  |  |  |  |
| temperature*mobility (t-14) | 0.000470*** | 0.000797*** |  |  |  |  |  |  |  |  |
|  | [0.000170] | [0.000240] |  |  |  |  |  |  |  |  |
| solar radiation (t-14) |  |  | -0.0537*** | -0.0469*** |  |  |  |  |  |  |
|  |  |  | [0.0188] | [0.0174] |  |  |  |  |  |  |
| solar radiation*mobility (t-14) |  |  | 0.000513** | 0.000653*** |  |  |  |  |  |  |
|  |  |  | [0.000190] | [0.000225] |  |  |  |  |  |  |
| humidity (t-14) |  |  |  |  | -0.0488*** | -0.0864*** |  |  |  |  |
|  |  |  |  |  | [0.0166] | [0.0209] |  |  |  |  |
| humidity*mobility (t-14) |  |  |  |  | 0.000415*** | 0.000789*** |  |  |  |  |
|  |  |  |  |  | [0.000149] | [0.000195] |  |  |  |  |
| precipitation (t-14) |  |  |  |  |  |  | 0.00151 | -0.0263* |  |  |
|  |  |  |  |  |  |  | [0.00922] | [0.0159] |  |  |
| precipitation*mobility (t-14) |  |  |  |  |  |  | 3.99e-06 | 0.000487 |  |  |
|  |  |  |  |  |  |  | [7.28e-05] | [0.000375] |  |  |
| wind speed (t-14) |  |  |  |  |  |  |  |  | 0.00669 | -0.0112 |
|  |  |  |  |  |  |  |  |  | [0.00936] | [0.0204] |
| wind speed *mobility (t-14) |  |  |  |  |  |  |  |  | -9.06e-05 | 0.000123 |
|  |  |  |  |  |  |  |  |  | [8.83e-05] | [0.000190] |
| Trend | Yes | Yes | Yes | Yes | Yes | Yes | Yes | Yes | Yes | Yes |
| Observations | 6,549 | 6,549 | 6,549 | 6,549 | 6,549 | 6,549 | 6,549 | 6,549 | 6,549 | 6,549 |
| Country | 37 | 37 | 37 | 37 | 37 | 37 | 37 | 37 | 37 | 37 |
| R-squared | 0.618 |  | 0.618 |  | 0.617 |  | 0.615 |  | 0.615 |  |

Note: the coefficients displayed are marginal effects. Standard errors (robust to within-country correlations for DFE) are reported in brackets. *** p < 0.01, ** < 0.05, * p < 0.1.

**Table F5. Direct effects of climate variables on Covid-19 infected cases: estimates until 15/09/2020 with alternative climate variables**

|  | **Covid-19 infected cases** | | | | | | | | | |
| --- | --- | --- | --- | --- | --- | --- | --- | --- | --- | --- |
|  | DFE | MG | DFE | MG | DFE | MG | DFE | MG | DFE | MG |
|  | (1) | (2) | (3) | (4) | (5) | (6) | (7) | (8) | (9) | (10) |
| casepop (t-1) | 0.389*** | 0.552*** | 0.388*** | 0.554*** | 0.390*** | 0.559*** | 0.390*** | 0.563*** | 0.390*** | 0.564*** |
|  | [0.127] | [0.0370] | [0.127] | [0.0370] | [0.127] | [0.0369] | [0.127] | [0.0364] | [0.127] | [0.0365] |
| casepop (t-7) | 0.578*** | 0.370*** | 0.579*** | 0.373*** | 0.580*** | 0.370*** | 0.579*** | 0.367*** | 0.579*** | 0.367*** |
|  | [0.144] | [0.0411] | [0.144] | [0.0408] | [0.144] | [0.0410] | [0.144] | [0.0406] | [0.144] | [0.0405] |
| temperature (t-7) | -0.105 | -0.321** |  |  |  |  |  |  |  |  |
|  | [0.0660] | [0.127] |  |  |  |  |  |  |  |  |
| solar radiation (t-7) |  |  | -0.136** | -0.242*** |  |  |  |  |  |  |
|  |  |  | [0.0571] | [0.0551] |  |  |  |  |  |  |
| humidity (t-7) |  |  |  |  | 0.0664 | 0.0598 |  |  |  |  |
|  |  |  |  |  | [0.0413] | [0.0385] |  |  |  |  |
| precipitation (t-7) |  |  |  |  |  |  | -0.0103 | -0.0143 |  |  |
|  |  |  |  |  |  |  | [0.0262] | [0.0239] |  |  |
| wind speed (t-7) |  |  |  |  |  |  |  |  | 0.0215 | -0.0115 |
|  |  |  |  |  |  |  |  |  | [0.0340] | [0.0409] |
| Trend | Yes | Yes | Yes | Yes | Yes | Yes | Yes | Yes | Yes | Yes |
| Observations | 8,396 | 8,396 | 8,396 | 8,396 | 8,396 | 8,396 | 8,396 | 8,396 | 8,396 | 8,396 |
| Country | 37 | 37 | 37 | 37 | 37 | 37 | 37 | 37 | 37 | 37 |
| R-squared | 0.786 |  | 0.786 |  | 0.786 |  | 0.786 |  | 0.786 |  |

Note: the coefficients displayed are marginal effects. Standard errors (robust to within-country correlations for DFE) are reported in brackets. *** p < 0.01, ** < 0.05, * p < 0.1.

**Table F6. Direct effects of climate variables on Covid-19 fatalities: estimates until 15/09/2020 with alternative climate variables**

|  | **Covid-19 fatalities** | | | | | | | | | |
| --- | --- | --- | --- | --- | --- | --- | --- | --- | --- | --- |
|  | DFE | MG | DFE | MG | DFE | MG | DFE | MG | DFE | MG |
|  | (1) | (2) | (3) | (4) | (5) | (6) | (7) | (8) | (9) | (10) |
| deathpop (t-1) | 0.644*** | 0.409*** | 0.646*** | 0.409*** | 0.647*** | 0.402*** | 0.647*** | 0.410*** | 0.647*** | 0.410*** |
|  | [0.105] | [0.0475] | [0.104] | [0.0483] | [0.104] | [0.0482] | [0.104] | [0.0475] | [0.104] | [0.0481] |
| deathpop (t-14) | 0.149*** | 0.122*** | 0.149*** | 0.111*** | 0.149*** | 0.114*** | 0.149*** | 0.118*** | 0.148*** | 0.119*** |
|  | [0.0407] | [0.0219] | [0.0414] | [0.0219] | [0.0414] | [0.0222] | [0.0413] | [0.0223] | [0.0413] | [0.0220] |
| casepop (t-14) | 0.00592*** | 0.0124*** | 0.00607*** | 0.0128*** | 0.00613** | 0.0122*** | 0.00613** | 0.0124*** | 0.00614** | 0.0128*** |
|  | [0.00213] | [0.00259] | [0.00219] | [0.00269] | [0.00226] | [0.00259] | [0.00227] | [0.00266] | [0.00228] | [0.00261] |
| temperature (t-14) | -0.0121** | -0.0135** |  |  |  |  |  |  |  |  |
|  | [0.00475] | [0.00528] |  |  |  |  |  |  |  |  |
| solar radiation (t-14) |  |  | -0.00578 | 0.00448 |  |  |  |  |  |  |
|  |  |  | [0.00345] | [0.00305] |  |  |  |  |  |  |
| humidity (t-14) |  |  |  |  | 3.00e-05 | -0.00429* |  |  |  |  |
|  |  |  |  |  | [0.00283] | [0.00226] |  |  |  |  |
| precipitation (t-14) |  |  |  |  |  |  | -0.000509 | 0.000274 |  |  |
|  |  |  |  |  |  |  | [0.00222] | [0.00257] |  |  |
| wind speed (t-14) |  |  |  |  |  |  |  |  | -0.000721 | -0.00134 |
|  |  |  |  |  |  |  |  |  | [0.00262] | [0.00363] |
| Trend | Yes | Yes | Yes | Yes | Yes | Yes | Yes | Yes | Yes | Yes |
| Observations | 7,880 | 7,880 | 7,880 | 7,880 | 7,880 | 7,880 | 7,880 | 7,880 | 7,880 | 7,880 |
| Country | 37 | 37 | 37 | 37 | 37 | 37 | 37 | 37 | 37 | 37 |
| R-squared | 0.610 |  | 0.609 |  | 0.609 |  | 0.609 |  | 0.609 |  |

Note: the coefficients displayed are marginal effects. Standard errors (robust to within-country correlations for DFE) are reported in brackets. *** p < 0.01, ** < 0.05, * p < 0.1.

**Table F7. Indirect effects of climate variables on Covid-19 infected cases through human mobility: estimates until 15/09/2020 with alternative climate variables**

|  | **Covid-19 infected cases** | | | | | | | | | |
| --- | --- | --- | --- | --- | --- | --- | --- | --- | --- | --- |
|  | DFE | MG | DFE | MG | DFE | MG | DFE | MG | DFE | MG |
|  | (1) | (2) | (3) | (4) | (5) | (6) | (7) | (8) | (9) | (10) |
| casepop (t-1) | 0.389*** | 0.502*** | 0.386*** | 0.509*** | 0.391*** | 0.532*** | 0.391*** | 0.542*** | 0.392*** | 0.542*** |
|  | [0.127] | [0.0396] | [0.127] | [0.0377] | [0.127] | [0.0390] | [0.127] | [0.0376] | [0.127] | [0.0373] |
| casepop (t-7) | 0.582*** | 0.359*** | 0.582*** | 0.368*** | 0.584*** | 0.365*** | 0.584*** | 0.365*** | 0.585*** | 0.365*** |
|  | [0.146] | [0.0433] | [0.146] | [0.0420] | [0.146] | [0.0425] | [0.147] | [0.0419] | [0.146] | [0.0418] |
| mobility (t-7) | 0.00231 | -0.000400 | 0.00157 | -0.000128 | 0.00177 | -0.000823 | 0.00207 | -0.000165 | 0.00225 | 0.00195 |
|  | [0.00140] | [0.00148] | [0.00130] | [0.00133] | [0.00128] | [0.000832] | [0.00134] | [0.00102] | [0.00140] | [0.00121] |
| temperature (t-7) | -0.244* | -0.840*** |  |  |  |  |  |  |  |  |
|  | [0.129] | [0.214] |  |  |  |  |  |  |  |  |
| temperature*mobility (t-7) | 0.000930 | 0.00490* |  |  |  |  |  |  |  |  |
|  | [0.00106] | [0.00252] |  |  |  |  |  |  |  |  |
| solar radiation (t-7) |  |  | -0.442** | -1.194*** |  |  |  |  |  |  |
|  |  |  | [0.167] | [0.264] |  |  |  |  |  |  |
| solar radiation*mobility (t-7) |  |  | 0.00273** | 0.00894*** |  |  |  |  |  |  |
|  |  |  | [0.00119] | [0.00242] |  |  |  |  |  |  |
| humidity (t-7) |  |  |  |  | 0.147 | 0.103 |  |  |  |  |
|  |  |  |  |  | [0.123] | [0.0810] |  |  |  |  |
| humidity*mobility (t-7) |  |  |  |  | -0.000921 | -0.000395 |  |  |  |  |
|  |  |  |  |  | [0.000976] | [0.000635] |  |  |  |  |
| precipitation (t-7) |  |  |  |  |  |  | 0.000594 | 0.0562 |  |  |
|  |  |  |  |  |  |  | [0.0928] | [0.0740] |  |  |
| precipitation*mobility (t-7) |  |  |  |  |  |  | -0.000101 | -0.000624 |  |  |
|  |  |  |  |  |  |  | [0.000662] | [0.000864] |  |  |
| wind speed (t-7) |  |  |  |  |  |  |  |  | -0.0781 | -0.165 |
|  |  |  |  |  |  |  |  |  | [0.124] | [0.123] |
| wind speed*mobility (t-7) |  |  |  |  |  |  |  |  | 0.000978 | 0.00175 |
|  |  |  |  |  |  |  |  |  | [0.000976] | [0.00110] |
| Trend | Yes | Yes | Yes | Yes | Yes | Yes | Yes | Yes | Yes | Yes |
| Observations | 8,322 | 8,322 | 8,322 | 8,322 | 8,322 | 8,322 | 8,322 | 8,322 | 8,322 | 8,322 |
| Country | 37 | 37 | 37 | 37 | 37 | 37 | 37 | 37 | 37 | 37 |
| R-squared | 0.786 |  | 0.787 |  | 0.786 |  | 0.786 |  | 0.786 |  |

Note: the coefficients displayed are marginal effects. Standard errors (robust to within-country correlations for DFE) are reported in brackets. *** p < 0.01, ** < 0.05, * p < 0.1.

**Table F8. Indirect effects of climate variables on Covid-19 fatalities through human mobility: estimates until 15/09/2020 with alternative climate variables**

|  | **Covid-19 fatalities** | | | | | | | | | |
| --- | --- | --- | --- | --- | --- | --- | --- | --- | --- | --- |
|  | DFE | MG | DFE | MG | DFE | MG | DFE | MG | DFE | MG |
|  | (1) | (2) | (3) | (4) | (5) | (6) | (7) | (8) | (9) | (10) |
| deathpop (t-1) | 0.667*** | 0.309*** | 0.667*** | 0.336*** | 0.671*** | 0.340*** | 0.671*** | 0.349*** | 0.670*** | 0.350*** |
|  | [0.112] | [0.0441] | [0.112] | [0.0457] | [0.112] | [0.0472] | [0.112] | [0.0471] | [0.112] | [0.0485] |
| deathpop (t-14) | 0.133*** | 0.0845*** | 0.131*** | 0.0783*** | 0.127*** | 0.0728*** | 0.128*** | 0.0721*** | 0.127*** | 0.0783*** |
|  | [0.0446] | [0.0243] | [0.0447] | [0.0243] | [0.0448] | [0.0249] | [0.0446] | [0.0247] | [0.0444] | [0.0239] |
| casepop (t-14) | 0.00473** | 0.0101*** | 0.00489** | 0.0116*** | 0.00536** | 0.0116*** | 0.00532** | 0.0116*** | 0.00529** | 0.0120*** |
|  | [0.00190] | [0.00251] | [0.00193] | [0.00272] | [0.00209] | [0.00260] | [0.00208] | [0.00266] | [0.00212] | [0.00269] |
| mobility (t-14) | -0.000273* | -0.00104*** | -0.000287** | -0.000692*** | -0.000243* | -0.000634*** | -0.000248** | -0.000630*** | -0.000273** | -0.000537*** |
|  | [0.000135] | [0.000301] | [0.000134] | [0.000173] | [0.000120] | [0.000131] | [0.000120] | [0.000127] | [0.000125] | [0.000132] |
| temperature (t-14) | -0.0279** | -0.0804*** |  |  |  |  |  |  |  |  |
|  | [0.0116] | [0.0241] |  |  |  |  |  |  |  |  |
| temperature*mobility (t-14) | 0.000186* | 0.000709*** |  |  |  |  |  |  |  |  |
|  | [0.000101] | [0.000240] |  |  |  |  |  |  |  |  |
| solar radiation (t-14) |  |  | -0.0250** | -0.0482*** |  |  |  |  |  |  |
|  |  |  | [0.00996] | [0.0150] |  |  |  |  |  |  |
| solar radiation*mobility (t-14) |  |  | 0.000181** | 0.000479*** |  |  |  |  |  |  |
|  |  |  | [8.26e-05] | [0.000142] |  |  |  |  |  |  |
| humidity (t-14) |  |  |  |  | -0.00512 | -0.00417 |  |  |  |  |
|  |  |  |  |  | [0.00618] | [0.00775] |  |  |  |  |
| humidity*mobility (t-14) |  |  |  |  | 6.18e-05 | 1.82e-05 |  |  |  |  |
|  |  |  |  |  | [5.52e-05] | [5.96e-05] |  |  |  |  |
| precipitation (t-14) |  |  |  |  |  |  | -0.00189 | -0.00505 |  |  |
|  |  |  |  |  |  |  | [0.00696] | [0.00817] |  |  |
| precipitation*mobility (t-14) |  |  |  |  |  |  | 1.76e-05 | 2.83e-05 |  |  |
|  |  |  |  |  |  |  | [5.09e-05] | [6.90e-05] |  |  |
| wind speed (t-14) |  |  |  |  |  |  |  |  | 0.0101 | 0.00279 |
|  |  |  |  |  |  |  |  |  | [0.00898] | [0.00789] |
| wind speed*mobility (t-14) |  |  |  |  |  |  |  |  | -0.000119 | -2.47e-05 |
|  |  |  |  |  |  |  |  |  | [7.72e-05] | [7.25e-05] |
| Trend | Yes | Yes | Yes | Yes | Yes | Yes | Yes | Yes | Yes | Yes |
| Observations | 7,806 | 7,806 | 7,806 | 7,806 | 7,806 | 7,806 | 7,806 | 7,806 | 7,806 | 7,806 |
| Country | 37 | 37 | 37 | 37 | 37 | 37 | 37 | 37 | 37 | 37 |
| R-squared | 0.642 |  | 0.642 |  | 0.642 |  | 0.641 |  | 0.642 |  |

Note: the coefficients displayed are marginal effects. Standard errors (robust to within-country correlations for DFE) are reported in brackets. *** p < 0.01, ** < 0.05, * p < 0.1.

**G. Accounting for potential outliers: estimates without Chile**

**Table G1.** **Direct effects of climate variables on Covid-19 infected cases**

|  | **Covid-19 infected cases** | | | | | | | | | |
| --- | --- | --- | --- | --- | --- | --- | --- | --- | --- | --- |
|  | DFE | MG | DFE | MG | DFE | MG | DFE | MG | DFE | MG |
|  | (1) | (2) | (3) | (4) | (5) | (6) | (7) | (8) | (9) | (10) |
| casepop (t-1) | 0.693*** | 0.614*** | 0.693*** | 0.616*** | 0.694*** | 0.614*** | 0.694*** | 0.625*** | 0.694*** | 0.627*** |
|  | [0.0373] | [0.0347] | [0.0374] | [0.0344] | [0.0370] | [0.0349] | [0.0371] | [0.0340] | [0.0371] | [0.0338] |
| casepop (t-7) | 0.243*** | 0.256*** | 0.246*** | 0.258*** | 0.243*** | 0.254*** | 0.243*** | 0.256*** | 0.243*** | 0.257*** |
|  | [0.0387] | [0.0307] | [0.0396] | [0.0306] | [0.0388] | [0.0304] | [0.0387] | [0.0306] | [0.0387] | [0.0308] |
| temperature (t-7) | -0.0856* | -0.208** |  |  |  |  |  |  |  |  |
|  | [0.0497] | [0.0882] |  |  |  |  |  |  |  |  |
| solar radiation (t-7) |  |  | -0.0976** | -0.0974 |  |  |  |  |  |  |
|  |  |  | [0.0480] | [0.0638] |  |  |  |  |  |  |
| humidity (t-7) |  |  |  |  | 0.0116 | -0.0492 |  |  |  |  |
|  |  |  |  |  | [0.0420] | [0.0563] |  |  |  |  |
| precipitation (t-7) |  |  |  |  |  |  | -0.0127 | -0.0224* |  |  |
|  |  |  |  |  |  |  | [0.0112] | [0.0129] |  |  |
| wind speed (t-7) |  |  |  |  |  |  |  |  | -0.0269* | -0.0573* |
|  |  |  |  |  |  |  |  |  | [0.0154] | [0.0308] |
| Trend | Yes | Yes | Yes | Yes | Yes | Yes | Yes | Yes | Yes | Yes |
| Observations | 6,264 | 6,264 | 6,264 | 6,264 | 6,264 | 6,264 | 6,264 | 6,264 | 6,264 | 6,264 |
| Country | 36 | 36 | 36 | 36 | 36 | 36 | 36 | 36 | 36 | 36 |
| R-squared | 0.791 |  | 0.791 |  | 0.790 |  | 0.790 |  | 0.790 |  |

Note: the coefficients displayed are marginal effects. Standard errors (robust to within-country correlations for DFE) are reported in brackets. *** p < 0.01, ** < 0.05, * p < 0.1.

**Table G2.** **Direct effects of climate variables on Covid-19 fatalities**

|  | **Covid-19 fatalities** | | | | | | | | | |
| --- | --- | --- | --- | --- | --- | --- | --- | --- | --- | --- |
|  | DFE | MG | DFE | MG | DFE | MG | DFE | MG | DFE | MG |
|  | (1) | (2) | (3) | (4) | (5) | (6) | (7) | (8) | (9) | (10) |
| deathpop (t-1) | 0.673*** | 0.375*** | 0.676*** | 0.378*** | 0.674*** | 0.369*** | 0.676*** | 0.383*** | 0.676*** | 0.380*** |
|  | [0.0888] | [0.0485] | [0.0886] | [0.0486] | [0.0890] | [0.0489] | [0.0886] | [0.0484] | [0.0886] | [0.0492] |
| deathpop (t-14) | 0.123*** | 0.108*** | 0.123*** | 0.103*** | 0.121*** | 0.0981*** | 0.122*** | 0.104*** | 0.121*** | 0.102*** |
|  | [0.0388] | [0.0261] | [0.0396] | [0.0260] | [0.0387] | [0.0261] | [0.0391] | [0.0258] | [0.0389] | [0.0258] |
| casepop (t-14) | 0.00688** | 0.0184*** | 0.00710** | 0.0186*** | 0.00695** | 0.0180*** | 0.00706** | 0.0185*** | 0.00709** | 0.0187*** |
|  | [0.00338] | [0.00421] | [0.00342] | [0.00433] | [0.00337] | [0.00416] | [0.00340] | [0.00429] | [0.00340] | [0.00429] |
| temperature (t-14) | -0.0147*** | -0.0210*** |  |  |  |  |  |  |  |  |
|  | [0.00438] | [0.00631] |  |  |  |  |  |  |  |  |
| solar radiation (t-14) |  |  | -0.00227 | 0.00641 |  |  |  |  |  |  |
|  |  |  | [0.00379] | [0.00536] |  |  |  |  |  |  |
| humidity (t-14) |  |  |  |  | -0.0107** | -0.0203*** |  |  |  |  |
|  |  |  |  |  | [0.00408] | [0.00572] |  |  |  |  |
| precipitation (t-14) |  |  |  |  |  |  | -0.00193 | -0.00324* |  |  |
|  |  |  |  |  |  |  | [0.00161] | [0.00171] |  |  |
| wind speed (t-14) |  |  |  |  |  |  |  |  | -0.00199 | 0.00410 |
|  |  |  |  |  |  |  |  |  | [0.00251] | [0.00455] |
| Trend | Yes | Yes | Yes | Yes | Yes | Yes | Yes | Yes | Yes | Yes |
| Observations | 5,760 | 5,760 | 5,760 | 5,760 | 5,760 | 5,760 | 5,760 | 5,760 | 5,760 | 5,760 |
| Country | 36 | 36 | 36 | 36 | 36 | 36 | 36 | 36 | 36 | 36 |
| R-squared | 0.638 |  | 0.638 |  | 0.638 |  | 0.638 |  | 0.638 |  |

Note: the coefficients displayed are marginal effects. Standard errors (robust to within-country correlations for DFE) are reported in brackets. *** p < 0.01, ** < 0.05, * p < 0.1.

**Table G3.** **Indirect effects of climate variables on Covid-19 infected cases through human mobility**

|  | **Covid-19 infected cases** | | | | | | | | | |
| --- | --- | --- | --- | --- | --- | --- | --- | --- | --- | --- |
|  | DFE | MG | DFE | MG | DFE | MG | DFE | MG | DFE | MG |
|  | (1) | (2) | (3) | (4) | (5) | (6) | (7) | (8) | (9) | (10) |
| casepop (t-1) | 0.689*** | 0.515*** | 0.687*** | 0.542*** | 0.691*** | 0.527*** | 0.694*** | 0.579*** | 0.694*** | 0.580*** |
|  | [0.0370] | [0.0412] | [0.0376] | [0.0367] | [0.0364] | [0.0419] | [0.0370] | [0.0369] | [0.0366] | [0.0370] |
| casepop (t-7) | 0.239*** | 0.223*** | 0.245*** | 0.244*** | 0.239*** | 0.219*** | 0.242*** | 0.234*** | 0.242*** | 0.239*** |
|  | [0.0397] | [0.0328] | [0.0404] | [0.0321] | [0.0395] | [0.0318] | [0.0393] | [0.0313] | [0.0394] | [0.0309] |
| mobility (t-7) | -0.000446 | -0.00239 | -0.00155* | -0.00516*** | -0.000545 | -0.00459*** | -5.31e-05 | -0.00272** | -8.69e-05 | -0.00307** |
|  | [0.000763] | [0.00374] | [0.000821] | [0.00148] | [0.000601] | [0.00132] | [0.000563] | [0.00128] | [0.000617] | [0.00127] |
| temperature (t-7) | -0.299*** | -0.760*** |  |  |  |  |  |  |  |  |
|  | [0.0970] | [0.229] |  |  |  |  |  |  |  |  |
| temperature*mobility (t-7) | 0.00207** | 0.00589* |  |  |  |  |  |  |  |  |
|  | [0.000957] | [0.00338] |  |  |  |  |  |  |  |  |
| solar radiation (t-7) |  |  | -0.401*** | -0.675*** |  |  |  |  |  |  |
|  |  |  | [0.141] | [0.205] |  |  |  |  |  |  |
| solar radiation*mobility (t-7) |  |  | 0.00297** | 0.00689*** |  |  |  |  |  |  |
|  |  |  | [0.00114] | [0.00211] |  |  |  |  |  |  |
| humidity (t-7) |  |  |  |  | -0.144* | -0.672*** |  |  |  |  |
|  |  |  |  |  | [0.0714] | [0.139] |  |  |  |  |
| humidity*mobility (t-7) |  |  |  |  | 0.00158** | 0.00685*** |  |  |  |  |
|  |  |  |  |  | [0.000648] | [0.00142] |  |  |  |  |
| precipitation (t-7) |  |  |  |  |  |  | -0.0483 | -0.0700 |  |  |
|  |  |  |  |  |  |  | [0.0359] | [0.0542] |  |  |
| precipitation*mobility (t-7) |  |  |  |  |  |  | 0.000373 | 0.000440 |  |  |
|  |  |  |  |  |  |  | [0.000349] | [0.000548] |  |  |
| wind speed (t-7) |  |  |  |  |  |  |  |  | -0.0168 | 0.141 |
|  |  |  |  |  |  |  |  |  | [0.0728] | [0.110] |
| wind speed *mobility (t-7) |  |  |  |  |  |  |  |  | -9.26e-05 | -0.00236* |
|  |  |  |  |  |  |  |  |  | [0.000623] | [0.00142] |
| Trend | Yes | Yes | Yes | Yes | Yes | Yes | Yes | Yes | Yes | Yes |
| Observations | 6,192 | 6,192 | 6,192 | 6,192 | 6,192 | 6,192 | 6,192 | 6,192 | 6,192 | 6,192 |
| Country | 36 | 36 | 36 | 36 | 36 | 36 | 36 | 36 | 36 | 36 |
| R-squared | 0.791 |  | 0.791 |  | 0.790 |  | 0.790 |  | 0.790 |  |

Note: the coefficients displayed are marginal effects. Standard errors (robust to within-country correlations for DFE) are reported in brackets. *** p < 0.01, ** < 0.05, * p < 0.1.

**Table G4.** **Indirect effects of climate variables on Covid-19 fatalities through human mobility**

|  | **Covid-19 fatalities** | | | | | | | | | |
| --- | --- | --- | --- | --- | --- | --- | --- | --- | --- | --- |
|  | DFE | MG | DFE | MG | DFE | MG | DFE | MG | DFE | MG |
|  | (1) | (2) | (3) | (4) | (5) | (6) | (7) | (8) | (9) | (10) |
| deathpop (t-1) | 0.697*** | 0.262*** | 0.698*** | 0.283*** | 0.698*** | 0.263*** | 0.706*** | 0.298*** | 0.706*** | 0.296*** |
|  | [0.0896] | [0.0452] | [0.0912] | [0.0458] | [0.0899] | [0.0460] | [0.0894] | [0.0470] | [0.0896] | [0.0499] |
| deathpop (t-14) | 0.111** | 0.0826*** | 0.109** | 0.0687** | 0.105** | 0.0732*** | 0.0993** | 0.0557** | 0.0995** | 0.0536* |
|  | [0.0421] | [0.0274] | [0.0433] | [0.0280] | [0.0416] | [0.0277] | [0.0414] | [0.0281] | [0.0415] | [0.0277] |
| casepop (t-14) | 0.00389 | 0.0113*** | 0.00463* | 0.0142*** | 0.00417 | 0.0110*** | 0.00537* | 0.0144*** | 0.00531* | 0.0147*** |
|  | [0.00258] | [0.00375] | [0.00258] | [0.00429] | [0.00258] | [0.00370] | [0.00269] | [0.00424] | [0.00269] | [0.00437] |
| mobility (t-14) | -0.000393** | -0.00117*** | -0.000496** | -0.00121*** | -0.000366** | -0.00111*** | -0.000318** | -0.00104*** | -0.000325** | -0.000961*** |
|  | [0.000154] | [0.000297] | [0.000189] | [0.000275] | [0.000142] | [0.000223] | [0.000123] | [0.000235] | [0.000124] | [0.000240] |
| temperature (t-14) | -0.0493*** | -0.0948*** |  |  |  |  |  |  |  |  |
|  | [0.0145] | [0.0261] |  |  |  |  |  |  |  |  |
| temperature*mobility (t-14) | 0.000408*** | 0.000949*** |  |  |  |  |  |  |  |  |
|  | [0.000138] | [0.000277] |  |  |  |  |  |  |  |  |
| solar radiation (t-14) |  |  | -0.0492** | -0.0566*** |  |  |  |  |  |  |
|  |  |  | [0.0193] | [0.0195] |  |  |  |  |  |  |
| solar radiation*mobility (t-14) |  |  | 0.000450** | 0.000768*** |  |  |  |  |  |  |
|  |  |  | [0.000180] | [0.000269] |  |  |  |  |  |  |
| humidity (t-14) |  |  |  |  | -0.0391*** | -0.0964*** |  |  |  |  |
|  |  |  |  |  | [0.0123] | [0.0234] |  |  |  |  |
| humidity*mobility (t-14) |  |  |  |  | 0.000353*** | 0.000911*** |  |  |  |  |
|  |  |  |  |  | [0.000117] | [0.000226] |  |  |  |  |
| precipitation (t-14) |  |  |  |  |  |  | -0.00656 | -0.0162 |  |  |
|  |  |  |  |  |  |  | [0.00454] | [0.0135] |  |  |
| precipitation*mobility (t-14) |  |  |  |  |  |  | 6.25e-05 | 0.000111 |  |  |
|  |  |  |  |  |  |  | [3.93e-05] | [0.000124] |  |  |
| wind speed (t-14) |  |  |  |  |  |  |  |  | 0.00463 | 0.00927 |
|  |  |  |  |  |  |  |  |  | [0.00754] | [0.0107] |
| wind speed*mobility (t-14) |  |  |  |  |  |  |  |  | -7.89e-05 | -8.43e-05 |
|  |  |  |  |  |  |  |  |  | [6.73e-05] | [9.72e-05] |
| Trend | Yes | Yes | Yes | Yes | Yes | Yes | Yes | Yes | Yes | Yes |
| Observations | 5,688 | 5,688 | 5,688 | 5,688 | 5,688 | 5,688 | 5,688 | 5,688 | 5,688 | 5,688 |
| Country | 36 | 36 | 36 | 36 | 36 | 36 | 36 | 36 | 36 | 36 |
| R-squared | 0.685 |  | 0.685 |  | 0.684 |  | 0.683 |  | 0.683 |  |

Note: the coefficients displayed are marginal effects. Standard errors (robust to within-country correlations for DFE) are reported in brackets. *** p < 0.01, ** < 0.05, * p < 0.1.

**H. Accounting for lockdown policies**

**Table H1. Indirect effects of climate variables on Covid-19 infected cases through human mobility: *Dummy_lockdown* variable**

|  | **Covid-19 infected cases** | | | | | | | | | |
| --- | --- | --- | --- | --- | --- | --- | --- | --- | --- | --- |
|  | DFE | MG | DFE | MG | DFE | MG | DFE | MG | DFE | MG |
|  | (1) | (2) | (3) | (4) | (5) | (6) | (7) | (8) | (9) | (10) |
| casepop (t-1) | 0.697*** | 0.491*** | 0.693*** | 0.517*** | 0.700*** | 0.501*** | 0.701*** | 0.547*** | 0.702*** | 0.546*** |
|  | [0.0354] | [0.0417] | [0.0358] | [0.0374] | [0.0350] | [0.0421] | [0.0352] | [0.0379] | [0.0349] | [0.0378] |
| casepop (t-7) | 0.239*** | 0.219*** | 0.243*** | 0.236*** | 0.241*** | 0.212*** | 0.243*** | 0.226*** | 0.243*** | 0.232*** |
|  | [0.0361] | [0.0323] | [0.0365] | [0.0319] | [0.0362] | [0.0314] | [0.0363] | [0.0312] | [0.0363] | [0.0309] |
| mobility (t-7) | 0.000105 | -0.00291 | -0.00115 | -0.00485*** | -8.49e-05 | -0.00575*** | 0.000253 | -0.00326** | 0.000270 | -0.00325** |
|  | [0.000929] | [0.00398] | [0.00106] | [0.00164] | [0.000805] | [0.00141] | [0.000686] | [0.00127] | [0.000695] | [0.00134] |
| dummy_lockdown (t-7) | 0.0734 | 0.108 | 0.0776 | 0.201** | 0.0602 | 0.173 | 0.0635 | 0.206* | 0.0659 | 0.199 |
|  | [0.102] | [0.103] | [0.0984] | [0.0967] | [0.0991] | [0.112] | [0.0950] | [0.119] | [0.0973] | [0.124] |
| temperature (t-7) | -0.279*** | -0.678*** |  |  |  |  |  |  |  |  |
|  | [0.0947] | [0.227] |  |  |  |  |  |  |  |  |
| temperature*mobility (t-7) | 0.00164 | 0.00500 |  |  |  |  |  |  |  |  |
|  | [0.000973] | [0.00346] |  |  |  |  |  |  |  |  |
| solar radiation (t-7) |  |  | -0.397*** | -0.580*** |  |  |  |  |  |  |
|  |  |  | [0.133] | [0.196] |  |  |  |  |  |  |
| solar radiation*mobility (t-7) |  |  | 0.00279** | 0.00613*** |  |  |  |  |  |  |
|  |  |  | [0.00112] | [0.00198] |  |  |  |  |  |  |
| humidity (t-7) |  |  |  |  | -0.130* | -0.684*** |  |  |  |  |
|  |  |  |  |  | [0.0667] | [0.143] |  |  |  |  |
| humidity*mobility (t-7) |  |  |  |  | 0.00125* | 0.00693*** |  |  |  |  |
|  |  |  |  |  | [0.000670] | [0.00141] |  |  |  |  |
| precipitation (t-7) |  |  |  |  |  |  | -0.0775 | -0.0713 |  |  |
|  |  |  |  |  |  |  | [0.0481] | [0.0502] |  |  |
| precipitation*mobility (t-7) |  |  |  |  |  |  | 0.000637 | 0.000283 |  |  |
|  |  |  |  |  |  |  | [0.000450] | [0.000561] |  |  |
| wind speed (t-7) |  |  |  |  |  |  |  |  | -0.0473 | 0.0775 |
|  |  |  |  |  |  |  |  |  | [0.0804] | [0.125] |
| wind speed*mobility (t-7) |  |  |  |  |  |  |  |  | 0.000221 | -0.00181 |
|  |  |  |  |  |  |  |  |  | [0.000691] | [0.00146] |
| Trend | Yes | Yes | Yes | Yes | Yes | Yes | Yes | Yes | Yes | Yes |
| Observations | 6,364 | 6,364 | 6,364 | 6,364 | 6,364 | 6,364 | 6,364 | 6,364 | 6,364 | 6,364 |
| Country | 37 | 37 | 37 | 37 | 37 | 37 | 37 | 37 | 37 | 37 |
| R-squared | 0.821 |  | 0.822 |  | 0.821 |  | 0.821 |  | 0.821 |  |

Note: the coefficients displayed are marginal effects. Standard errors (robust to within-country correlations for DFE) are reported in brackets. *** p < 0.01, ** < 0.05, * p < 0.1. *Dummy_lockdown* is equal to 1 if day t is associated with a lockdown period, and 0 (non-lockdown period otherwise).

**Table H2. Indirect effects of climate variables on Covid-19 fatalities through human mobility: *Dummy_lockdown* variable**

|  | **Covid-19 fatalities** | | | | | | | | | |
| --- | --- | --- | --- | --- | --- | --- | --- | --- | --- | --- |
|  | DFE | MG | DFE | MG | DFE | MG | DFE | MG | DFE | MG |
|  | (1) | (2) | (3) | (4) | (5) | (6) | (7) | (8) | (9) | (10) |
| deathpop (t-1) | 0.594*** | 0.227*** | 0.596*** | 0.242*** | 0.595*** | 0.226*** | 0.605*** | 0.254*** | 0.603*** | 0.248*** |
|  | [0.120] | [0.0454] | [0.120] | [0.0457] | [0.120] | [0.0458] | [0.119] | [0.0470] | [0.120] | [0.0493] |
| deathpop (t-14) | 0.138*** | 0.0871*** | 0.137** | 0.0740*** | 0.132** | 0.0781*** | 0.126** | 0.0634** | 0.126** | 0.0637** |
|  | [0.0495] | [0.0269] | [0.0506] | [0.0268] | [0.0488] | [0.0272] | [0.0490] | [0.0273] | [0.0489] | [0.0267] |
| casepop (t-14) | 0.00519* | 0.0106*** | 0.00581** | 0.0132*** | 0.00575* | 0.0102*** | 0.00699** | 0.0131*** | 0.00695** | 0.0133*** |
|  | [0.00300] | [0.00357] | [0.00282] | [0.00418] | [0.00312] | [0.00352] | [0.00311] | [0.00405] | [0.00320] | [0.00416] |
| mobility (t-14) | -0.000336* | -0.00117*** | -0.000479** | -0.00120*** | -0.000306** | -0.00108*** | -0.000256* | -0.000760** | -0.000266* | -0.000786*** |
|  | [0.000168] | [0.000296] | [0.000202] | [0.000265] | [0.000151] | [0.000218] | [0.000132] | [0.000319] | [0.000136] | [0.000268] |
| dummy_lockdown (t-14) | 0.0370** | 0.00696 | 0.0362** | 0.0116 | 0.0353** | 0.0134* | 0.0369** | 0.0226*** | 0.0371*** | 0.0188** |
|  | [0.0139] | [0.00666] | [0.0134] | [0.00712] | [0.0133] | [0.00691] | [0.0136] | [0.00829] | [0.0131] | [0.00795] |
| temperature (t-14) | -0.0629*** | -0.0871*** |  |  |  |  |  |  |  |  |
|  | [0.0195] | [0.0265] |  |  |  |  |  |  |  |  |
| temperature*mobility (t-14) | 0.000497*** | 0.000910*** |  |  |  |  |  |  |  |  |
|  | [0.000169] | [0.000275] |  |  |  |  |  |  |  |  |
| solar radiation (t-14) |  |  | -0.0606*** | -0.0531*** |  |  |  |  |  |  |
|  |  |  | [0.0204] | [0.0194] |  |  |  |  |  |  |
| solar radiation*mobility (t-14) |  |  | 0.000552*** | 0.000761*** |  |  |  |  |  |  |
|  |  |  | [0.000194] | [0.000263] |  |  |  |  |  |  |
| humidity (t-14) |  |  |  |  | -0.0507*** | -0.0905*** |  |  |  |  |
|  |  |  |  |  | [0.0168] | [0.0229] |  |  |  |  |
| humidity*mobility (t-14) |  |  |  |  | 0.000436*** | 0.000851*** |  |  |  |  |
|  |  |  |  |  | [0.000144] | [0.000215] |  |  |  |  |
| precipitation (t-14) |  |  |  |  |  |  | 0.00334 | -0.0277 |  |  |
|  |  |  |  |  |  |  | [0.0108] | [0.0169] |  |  |
| precipitation*mobility (t-14) |  |  |  |  |  |  | -1.11e-05 | 0.000496 |  |  |
|  |  |  |  |  |  |  | [8.38e-05] | [0.000386] |  |  |
| wind speed (t-14) |  |  |  |  |  |  |  |  | 0.00829 | -0.00905 |
|  |  |  |  |  |  |  |  |  | [0.00923] | [0.0216] |
| wind speed*mobility (t-14) |  |  |  |  |  |  |  |  | -0.000123 | 7.49e-05 |
|  |  |  |  |  |  |  |  |  | [8.86e-05] | [0.000195] |
| Trend | Yes | Yes | Yes | Yes | Yes | Yes | Yes | Yes | Yes | Yes |
| Observations | 5,846 | 5,846 | 5,846 | 5,846 | 5,846 | 5,846 | 5,846 | 5,846 | 5,846 | 5,846 |
| Country | 37 | 37 | 37 | 37 | 37 | 37 | 37 | 37 | 37 | 37 |
| R-squared | 0.602 |  | 0.602 |  | 0.601 |  | 0.598 |  | 0.599 |  |

Note: the coefficients displayed are marginal effects. Standard errors (robust to within-country correlations for DFE) are reported in brackets. *** p < 0.01, ** < 0.05, * p < 0.1. *Dummy_lockdown* is equal to 1 if day t is associated with a lockdown period, and 0 (non-lockdown period otherwise).

**Table H3. Indirect effects of climate variables on Covid-19 infected cases through human mobility: *Time_lockdown* variable**

|  | **Covid-19 infected cases** | | | | | | | | | |
| --- | --- | --- | --- | --- | --- | --- | --- | --- | --- | --- |
|  | DFE | MG | DFE | MG | DFE | MG | DFE | MG | DFE | MG |
|  | (1) | (2) | (3) | (4) | (5) | (6) | (7) | (8) | (9) | (10) |
| casepop (t-1) | 0.697*** | 0.492*** | 0.693*** | 0.520*** | 0.700*** | 0.504*** | 0.701*** | 0.551*** | 0.702*** | 0.552*** |
|  | [0.0357] | [0.0416] | [0.0362] | [0.0370] | [0.0353] | [0.0420] | [0.0357] | [0.0372] | [0.0354] | [0.0372] |
| casepop (t-7) | 0.240*** | 0.234*** | 0.244*** | 0.255*** | 0.242*** | 0.231*** | 0.244*** | 0.246*** | 0.244*** | 0.249*** |
|  | [0.0364] | [0.0325] | [0.0368] | [0.0326] | [0.0365] | [0.0314] | [0.0364] | [0.0312] | [0.0364] | [0.0306] |
| mobility (t-7) | -0.000330 | -0.00464 | -0.00161* | -0.00711*** | -0.000484 | -0.00690*** | -0.000177 | -0.00550*** | -0.000166 | -0.00548*** |
|  | [0.000803] | [0.00388] | [0.000940] | [0.00178] | [0.000712] | [0.00145] | [0.000607] | [0.00135] | [0.000647] | [0.00130] |
| time_lockdown (t-7) | -0.000179 | -0.00437 | -0.000365 | -0.00436 | -0.000470 | -0.00434 | -0.000679 | -0.00569 | -0.000665 | -0.00550 |
|  | [0.00250] | [0.00623] | [0.00251] | [0.00586] | [0.00266] | [0.00602] | [0.00261] | [0.00567] | [0.00265] | [0.00581] |
| temperature (t-7) | -0.278*** | -0.691*** |  |  |  |  |  |  |  |  |
|  | [0.0934] | [0.205] |  |  |  |  |  |  |  |  |
| temperature*mobility (t-7) | 0.00166* | 0.00527* |  |  |  |  |  |  |  |  |
|  | [0.000918] | [0.00320] |  |  |  |  |  |  |  |  |
| solar radiation (t-7) |  |  | -0.396*** | -0.579*** |  |  |  |  |  |  |
|  |  |  | [0.130] | [0.190] |  |  |  |  |  |  |
| solar radiation*mobility (t-7) |  |  | 0.00280** | 0.00601*** |  |  |  |  |  |  |
|  |  |  | [0.00107] | [0.00190] |  |  |  |  |  |  |
| humidity (t-7) |  |  |  |  | -0.132* | -0.646*** |  |  |  |  |
|  |  |  |  |  | [0.0712] | [0.127] |  |  |  |  |
| humidity*mobility (t-7) |  |  |  |  | 0.00128* | 0.00657*** |  |  |  |  |
|  |  |  |  |  | [0.000654] | [0.00130] |  |  |  |  |
| precipitation (t-7) |  |  |  |  |  |  | -0.0815 | -0.0511 |  |  |
|  |  |  |  |  |  |  | [0.0494] | [0.0573] |  |  |
| precipitation*mobility (t-7) |  |  |  |  |  |  | 0.000668 | 5.10e-05 |  |  |
|  |  |  |  |  |  |  | [0.000454] | [0.000624] |  |  |
| wind speed (t-7) |  |  |  |  |  |  |  |  | -0.0518 | 0.0730 |
|  |  |  |  |  |  |  |  |  | [0.0805] | [0.116] |
| wind speed*mobility (t-7) |  |  |  |  |  |  |  |  | 0.000253 | -0.00173 |
|  |  |  |  |  |  |  |  |  | [0.000692] | [0.00138] |
| Trend | Yes | Yes | Yes | Yes | Yes | Yes | Yes | Yes | Yes | Yes |
| Observations | 6,364 | 6,364 | 6,364 | 6,364 | 6,364 | 6,364 | 6,364 | 6,364 | 6,364 | 6,364 |
| Country | 37 | 37 | 37 | 37 | 37 | 37 | 37 | 37 | 37 | 37 |
| R-squared | 0.821 |  | 0.822 |  | 0.821 |  | 0.821 |  | 0.821 |  |

Note: the coefficients displayed are marginal effects. Standard errors (robust to within-country correlations for DFE) are reported in brackets. *** p < 0.01, ** < 0.05, * p < 0.1. *Time_lockdown* corresponds to the number of days since a lockdown policy has been implemented.

**Table H4. Indirect effects of climate variables on Covid-19 fatalities through human mobility: *Time_lockdown* variable**

|  | **Covid-19 fatalities** | | | | | | | | | |
| --- | --- | --- | --- | --- | --- | --- | --- | --- | --- | --- |
|  | DFE | MG | DFE | MG | DFE | MG | DFE | MG | DFE | MG |
|  | (1) | (2) | (3) | (4) | (5) | (6) | (7) | (8) | (9) | (10) |
| deathpop (t-1) | 0.601*** | 0.234*** | 0.603*** | 0.253*** | 0.602*** | 0.236*** | 0.612*** | 0.267*** | 0.610*** | 0.263*** |
|  | [0.120] | [0.0453] | [0.121] | [0.0457] | [0.121] | [0.0459] | [0.120] | [0.0472] | [0.121] | [0.0495] |
| deathpop (t-14) | 0.142*** | 0.0842*** | 0.141*** | 0.0694** | 0.136*** | 0.0746*** | 0.131** | 0.0596** | 0.131** | 0.0570** |
|  | [0.0494] | [0.0267] | [0.0505] | [0.0271] | [0.0487] | [0.0271] | [0.0490] | [0.0273] | [0.0487] | [0.0270] |
| casepop (t-14) | 0.00521* | 0.0112*** | 0.00584** | 0.0137*** | 0.00573* | 0.0111*** | 0.00701** | 0.0140*** | 0.00698** | 0.0140*** |
|  | [0.00290] | [0.00333] | [0.00274] | [0.00392] | [0.00300] | [0.00336] | [0.00302] | [0.00394] | [0.00308] | [0.00395] |
| mobility (t-14) | -0.000527** | -0.00128*** | -0.000665*** | -0.00134*** | -0.000490** | -0.00119*** | -0.000449** | -0.000985*** | -0.000457** | -0.000992*** |
|  | [0.000202] | [0.000335] | [0.000238] | [0.000316] | [0.000182] | [0.000260] | [0.000169] | [0.000357] | [0.000169] | [0.000315] |
| time_lockdown (t-14) | 0.000120 | -0.000133 | 6.07e-05 | -6.16e-05 | 8.28e-05 | -0.000104 | 5.78e-06 | -7.54e-05 | 1.55e-05 | -0.000155 |
|  | [0.000204] | [0.000313] | [0.000216] | [0.000326] | [0.000212] | [0.000300] | [0.000219] | [0.000354] | [0.000220] | [0.000328] |
| temperature (t-14) | -0.0629*** | -0.0867*** |  |  |  |  |  |  |  |  |
|  | [0.0194] | [0.0252] |  |  |  |  |  |  |  |  |
| temperature*mobility (t-14) | 0.000516*** | 0.000905*** |  |  |  |  |  |  |  |  |
|  | [0.000177] | [0.000261] |  |  |  |  |  |  |  |  |
| solar radiation (t-14) |  |  | -0.0608*** | -0.0524*** |  |  |  |  |  |  |
|  |  |  | [0.0205] | [0.0190] |  |  |  |  |  |  |
| solar radiation*mobility (t-14) |  |  | 0.000562*** | 0.000732*** |  |  |  |  |  |  |
|  |  |  | [0.000200] | [0.000254] |  |  |  |  |  |  |
| humidity (t-14) |  |  |  |  | -0.0516*** | -0.0892*** |  |  |  |  |
|  |  |  |  |  | [0.0170] | [0.0219] |  |  |  |  |
| humidity*mobility (t-14) |  |  |  |  | 0.000456*** | 0.000851*** |  |  |  |  |
|  |  |  |  |  | [0.000153] | [0.000214] |  |  |  |  |
| precipitation (t-14) |  |  |  |  |  |  | 0.00173 | -0.0234 |  |  |
|  |  |  |  |  |  |  | [0.00964] | [0.0166] |  |  |
| precipitation*mobility (t-14) |  |  |  |  |  |  | 1.38e-06 | 0.000450 |  |  |
|  |  |  |  |  |  |  | [7.53e-05] | [0.000396] |  |  |
| wind speed (t-14) |  |  |  |  |  |  |  |  | 0.00610 | -0.0104 |
|  |  |  |  |  |  |  |  |  | [0.00958] | [0.0211] |
| wind speed*mobility (t-14) |  |  |  |  |  |  |  |  | -0.000105 | 8.88e-05 |
|  |  |  |  |  |  |  |  |  | [9.09e-05] | [0.000188] |
| Trend | Yes | Yes | Yes | Yes | Yes | Yes | Yes | Yes | Yes | Yes |
| Observations | 5,846 | 5,846 | 5,846 | 5,846 | 5,846 | 5,846 | 5,846 | 5,846 | 5,846 | 5,846 |
| Country | 0.600 |  | 0.600 |  | 0.599 |  | 0.597 |  | 0.597 |  |
| R-squared | 37 | 37 | 37 | 37 | 37 | 37 | 37 | 37 | 37 | 37 |

Note: the coefficients displayed are marginal effects. Standard errors (robust to within-country correlations for DFE) are reported in brackets. *** p < 0.01, ** < 0.05, * p < 0.1. *Time_lockdown* corresponds to the number of days since a lockdown policy has been implemented.

**I. Accounting for non-standardized climate variables**

**I1. Direct effects of climate variables on Covid-19 infected cases**

|  | **Covid-19 infected cases** | | | | | | | | | |
| --- | --- | --- | --- | --- | --- | --- | --- | --- | --- | --- |
|  | DFE | MG | DFE | MG | DFE | MG | DFE | MG | DFE | MG |
|  | (1) | (2) | (3) | (4) | (5) | (6) | (7) | (8) | (9) | (10) |
| casepop (t-1) | 0.700*** | 0.618*** | 0.699*** | 0.620*** | 0.702*** | 0.618*** | 0.702*** | 0.629*** | 0.702*** | 0.629*** |
|  | [0.0357] | [0.0340] | [0.0359] | [0.0336] | [0.0356] | [0.0342] | [0.0357] | [0.0333] | [0.0356] | [0.0330] |
| casepop (t-7) | 0.242*** | 0.255*** | 0.246*** | 0.257*** | 0.244*** | 0.253*** | 0.244*** | 0.256*** | 0.244*** | 0.256*** |
|  | [0.0355] | [0.0299] | [0.0359] | [0.0298] | [0.0357] | [0.0296] | [0.0356] | [0.0298] | [0.0356] | [0.0299] |
| temperature (t-7) | -0.0145** | -0.0293** |  |  |  |  |  |  |  |  |
|  | [0.00691] | [0.0120] |  |  |  |  |  |  |  |  |
| solar radiation (t-7) |  |  | -0.00136** | -0.00129* |  |  |  |  |  |  |
|  |  |  | [0.000523] | [0.000762] |  |  |  |  |  |  |
| humidity (t-7) |  |  |  |  | -1.570 | -25.15 |  |  |  |  |
|  |  |  |  |  | [13.65] | [20.04] |  |  |  |  |
| precipitation (t-7) |  |  |  |  |  |  | -363.9 | -732.7* |  |  |
|  |  |  |  |  |  |  | [246.6] | [381.3] |  |  |
| wind speed (t-7) |  |  |  |  |  |  |  |  | -0.0161* | -0.0624* |
|  |  |  |  |  |  |  |  |  | [0.00887] | [0.0344] |
| Trend | Yes | Yes | Yes | Yes | Yes | Yes | Yes | Yes | Yes | Yes |
| Observations | 6,438 | 6,438 | 6,438 | 6,438 | 6,438 | 6,438 | 6,438 | 6,438 | 6,438 | 6,438 |
| Country | 37 | 37 | 37 | 37 | 37 | 37 | 37 | 37 | 37 | 37 |
| R-squared | 0.821 |  | 0.821 |  | 0.821 |  | 0.821 |  | 0.821 |  |

Note: the coefficients displayed are marginal effects. Standard errors (robust to within-country correlations for DFE) are reported in brackets. *** p < 0.01, ** < 0.05, * p < 0.1.

**I2. Direct effects of climate variables on Covid-19 fatalities**

|  | **Covid-19 infected cases** | | | | | | | | | |
| --- | --- | --- | --- | --- | --- | --- | --- | --- | --- | --- |
|  | DFE | MG | DFE | MG | DFE | MG | DFE | MG | DFE | MG |
|  | (1) | (2) | (3) | (4) | (5) | (6) | (7) | (8) | (9) | (10) |
| deathpop (t-1) | 0.590*** | 0.365*** | 0.593*** | 0.367*** | 0.590*** | 0.359*** | 0.593*** | 0.373*** | 0.593*** | 0.369*** |
|  | [0.111] | [0.0482] | [0.111] | [0.0484] | [0.112] | [0.0486] | [0.111] | [0.0480] | [0.111] | [0.0490] |
| deathpop (t-14) | 0.155*** | 0.107*** | 0.154*** | 0.102*** | 0.152*** | 0.0974*** | 0.153*** | 0.102*** | 0.152*** | 0.103*** |
|  | [0.0468] | [0.0254] | [0.0477] | [0.0253] | [0.0460] | [0.0254] | [0.0468] | [0.0251] | [0.0463] | [0.0251] |
| casepop (t-14) | 0.00806** | 0.0185*** | 0.00857** | 0.0187*** | 0.00832** | 0.0181*** | 0.00865** | 0.0185*** | 0.00868** | 0.0188*** |
|  | [0.00327] | [0.00409] | [0.00341] | [0.00421] | [0.00331] | [0.00405] | [0.00344] | [0.00417] | [0.00344] | [0.00417] |
| temperature (t-14) | -0.00250*** | -0.00235** |  |  |  |  |  |  |  |  |
|  | [0.000747] | [0.00105] |  |  |  |  |  |  |  |  |
| solar radiation (t-14) |  |  | -3.60e-05 | 0.000100 |  |  |  |  |  |  |
|  |  |  | [4.68e-05] | [6.71e-05] |  |  |  |  |  |  |
| humidity (t-14) |  |  |  |  | -4.648** | -6.347*** |  |  |  |  |
|  |  |  |  |  | [1.726] | [1.833] |  |  |  |  |
| precipitation (t-14) |  |  |  |  |  |  | 6.910 | 4.926 |  |  |
|  |  |  |  |  |  |  | [54.33] | [76.51] |  |  |
| wind speed (t-14) |  |  |  |  |  |  |  |  | -0.00173 | -0.00215 |
|  |  |  |  |  |  |  |  |  | [0.00162] | [0.00514] |
| Trend | Yes | Yes | Yes | Yes | Yes | Yes | Yes | Yes | Yes | Yes |
| Observations | 5,920 | 5,920 | 5,920 | 5,920 | 5,920 | 5,920 | 5,920 | 5,920 | 5,920 | 5,920 |
| Country | 37 | 37 | 37 | 37 | 37 | 37 | 37 | 37 | 37 | 37 |
| R-squared | 0.563 |  | 0.562 |  | 0.563 |  | 0.562 |  | 0.562 |  |

Note: the coefficients displayed are marginal effects. Standard errors (robust to within-country correlations for DFE) are reported in brackets. *** p < 0.01, ** < 0.05, * p < 0.1.

**I3. Indirect effects of climate variables on Covid-19 infected cases through human mobility**

|  | **Covid-19 infected cases** | | | | | | | | | |
| --- | --- | --- | --- | --- | --- | --- | --- | --- | --- | --- |
|  | DFE | MG | DFE | MG | DFE | MG | DFE | MG | DFE | MG |
|  | (1) | (2) | (3) | (4) | (5) | (6) | (7) | (8) | (9) | (10) |
| casepop (t-1) | 0.697*** | 0.521*** | 0.693*** | 0.548*** | 0.700*** | 0.533*** | 0.701*** | 0.583*** | 0.702*** | 0.583*** |
|  | [0.0357] | [0.0406] | [0.0361] | [0.0361] | [0.0352] | [0.0411] | [0.0355] | [0.0361] | [0.0352] | [0.0362] |
| casepop (t-7) | 0.240*** | 0.223*** | 0.243*** | 0.244*** | 0.241*** | 0.219*** | 0.244*** | 0.235*** | 0.244*** | 0.240*** |
|  | [0.0360] | [0.0319] | [0.0363] | [0.0312] | [0.0361] | [0.0309] | [0.0361] | [0.0304] | [0.0360] | [0.0301] |
| mobility (t-7) | -0.00299 | -0.0117 | -0.00691** | -0.0179*** | -0.00333* | -0.0209*** | -0.000474 | -0.00314** | -0.000581 | 0.000531 |
|  | [0.00204] | [0.00855] | [0.00276] | [0.00479] | [0.00181] | [0.00394] | [0.000545] | [0.00127] | [0.00131] | [0.00296] |
| temperature (t-7) | -0.0392*** | -0.103*** |  |  |  |  |  |  |  |  |
|  | [0.0135] | [0.0315] |  |  |  |  |  |  |  |  |
| temperature*mobility (t-7) | 0.000235* | 0.000797* |  |  |  |  |  |  |  |  |
|  | [0.000138] | [0.000462] |  |  |  |  |  |  |  |  |
| solar radiation (t-7) |  |  | -0.00483*** | -0.00791*** |  |  |  |  |  |  |
|  |  |  | [0.00163] | [0.00245] |  |  |  |  |  |  |
| solar radiation*mobility (t-7) |  |  | 3.43e-05** | 8.07e-05*** |  |  |  |  |  |  |
|  |  |  | [1.37e-05] | [2.52e-05] |  |  |  |  |  |  |
| humidity (t-7) |  |  |  |  | -43.75* | -232.4*** |  |  |  |  |
|  |  |  |  |  | [22.22] | [46.09] |  |  |  |  |
| humidity*mobility (t-7) |  |  |  |  | 0.423* | 2.322*** |  |  |  |  |
|  |  |  |  |  | [0.218] | [0.458] |  |  |  |  |
| precipitation (t-7) |  |  |  |  |  |  | -1,598 | -1,488 |  |  |
|  |  |  |  |  |  |  | [993.8] | [1,048] |  |  |
| precipitation*mobility (t-7) |  |  |  |  |  |  | 13.06 | 4.797 |  |  |
|  |  |  |  |  |  |  | [9.215] | [11.27] |  |  |
| wind speed (t-7) |  |  |  |  |  |  |  |  | -0.0286 | 0.0293 |
|  |  |  |  |  |  |  |  |  | [0.0455] | [0.0792] |
| wind speed*mobility (t-7) |  |  |  |  |  |  |  |  | 0.000136 | -0.000885 |
|  |  |  |  |  |  |  |  |  | [0.000392] | [0.000906] |
| Trend | Yes | Yes | Yes | Yes | Yes | Yes | Yes | Yes | Yes | Yes |
| Observations | 6,364 | 6,364 | 6,364 | 6,364 | 6,364 | 6,364 | 6,364 | 6,364 | 6,364 | 6,364 |
| Country | 37 | 37 | 37 | 37 | 37 | 37 | 37 | 37 | 37 | 37 |
| R-squared | 0.821 |  | 0.822 |  | 0.821 |  | 0.821 |  | 0.821 |  |

Note: the coefficients displayed are marginal effects. Standard errors (robust to within-country correlations for DFE) are reported in brackets. *** p < 0.01, ** < 0.05, * p < 0.1.

**I4. Indirect effects of climate variables on Covid-19 fatalities through human mobility**

|  | **Covid-19 fatalities** | | | | | | | | | |
| --- | --- | --- | --- | --- | --- | --- | --- | --- | --- | --- |
|  | DFE | MG | DFE | MG | DFE | MG | DFE | MG | DFE | MG |
|  | (1) | (2) | (3) | (4) | (5) | (6) | (7) | (8) | (9) | (10) |
| deathpop (t-1) | 0.601*** | 0.255*** | 0.603*** | 0.276*** | 0.602*** | 0.256*** | 0.612*** | 0.290*** | 0.610*** | 0.287*** |
|  | [0.120] | [0.0446] | [0.121] | [0.0453] | [0.120] | [0.0453] | [0.120] | [0.0464] | [0.121] | [0.0492] |
| deathpop (t-14) | 0.144*** | 0.0819*** | 0.142*** | 0.0683** | 0.137*** | 0.0727*** | 0.131** | 0.0550** | 0.131** | 0.0552** |
|  | [0.0500] | [0.0267] | [0.0510] | [0.0272] | [0.0492] | [0.0269] | [0.0496] | [0.0273] | [0.0492] | [0.0270] |
| casepop (t-14) | 0.00523* | 0.0116*** | 0.00584** | 0.0144*** | 0.00574* | 0.0113*** | 0.00701** | 0.0146*** | 0.00698** | 0.0148*** |
|  | [0.00292] | [0.00366] | [0.00274] | [0.00418] | [0.00300] | [0.00361] | [0.00301] | [0.00413] | [0.00308] | [0.00425] |
| mobility (t-14) | -0.00135*** | -0.00262*** | -0.00173*** | -0.00265*** | -0.00151*** | -0.00305*** | -0.000451*** | -0.00111*** | -0.000238 | -0.000941*** |
|  | [0.000469] | [0.000696] | [0.000607] | [0.000714] | [0.000504] | [0.000699] | [0.000145] | [0.000245] | [0.000200] | [0.000344] |
| temperature (t-14) | -0.00870*** | -0.0126*** |  |  |  |  |  |  |  |  |
|  | [0.00269] | [0.00363] |  |  |  |  |  |  |  |  |
| temperature*mobility (t-14) | 7.13e-05*** | 0.000131*** |  |  |  |  |  |  |  |  |
|  | [2.49e-05] | [3.78e-05] |  |  |  |  |  |  |  |  |
| solar radiation (t-14) |  |  | -0.000737*** | -0.000674*** |  |  |  |  |  |  |
|  |  |  | [0.000249] | [0.000231] |  |  |  |  |  |  |
| solar radiation*mobility (t-14) |  |  | 6.81e-06*** | 9.45e-06*** |  |  |  |  |  |  |
|  |  |  | [2.43e-06] | [3.18e-06] |  |  |  |  |  |  |
| humidity (t-14) |  |  |  |  | -16.69*** | -30.49*** |  |  |  |  |
|  |  |  |  |  | [5.572] | [7.461] |  |  |  |  |
| humidity*mobility (t-14) |  |  |  |  | 0.147*** | 0.290*** |  |  |  |  |
|  |  |  |  |  | [0.0506] | [0.0719] |  |  |  |  |
| precipitation (t-14) |  |  |  |  |  |  | 34.18 | -528.1 |  |  |
|  |  |  |  |  |  |  | [189.0] | [332.1] |  |  |
| precipitation*mobility (t-14) |  |  |  |  |  |  | 0.0284 | 9.457 |  |  |
|  |  |  |  |  |  |  | [1.474] | [7.644] |  |  |
| wind speed (t-14) |  |  |  |  |  |  |  |  | 0.00346 | -0.00458 |
|  |  |  |  |  |  |  |  |  | [0.00543] | [0.0115] |
| wind speed*mobility (t-14) |  |  |  |  |  |  |  |  | -5.95e-05 | 3.87e-05 |
|  |  |  |  |  |  |  |  |  | [5.15e-05] | [0.000102] |
| Trend |  |  |  |  |  |  |  |  |  |  |
| Observations | 5,846 | 5,846 | 5,846 | 5,846 | 5,846 | 5,846 | 5,846 | 5,846 | 5,846 | 5,846 |
| Country | 37 | 37 | 37 | 37 | 37 | 37 | 37 | 37 | 37 | 37 |
| R-squared | 0.600 |  | 0.600 |  | 0.599 |  | 0.597 |  | 0.597 |  |

Note: the coefficients displayed are marginal effects. Standard errors (robust to within-country correlations for DFE) are reported in brackets. *** p < 0.01, ** < 0.05, * p < 0.1.

**J. Accounting for seasonality in climate variables**

**J1. Direct effects of climate variables on Covid-19 infected cases: *season index***

|  | **Covid-19 infected cases** | | | | | | | | | |
| --- | --- | --- | --- | --- | --- | --- | --- | --- | --- | --- |
|  | DFE | MG | DFE | MG | DFE | MG | DFE | MG | DFE | MG |
|  | (1) | (2) | (3) | (4) | (5) | (6) | (7) | (8) | (9) | (10) |
| casepop (t-1) | 0.697*** | 0.600*** | 0.696*** | 0.603*** | 0.701*** | 0.599*** | 0.701*** | 0.610*** | 0.701*** | 0.611*** |
|  | [0.0366] | [0.0337] | [0.0368] | [0.0332] | [0.0361] | [0.0339] | [0.0361] | [0.0328] | [0.0361] | [0.0326] |
| casepop (t-7) | 0.246*** | 0.270*** | 0.252*** | 0.272*** | 0.246*** | 0.267*** | 0.247*** | 0.270*** | 0.247*** | 0.270*** |
|  | [0.0370] | [0.0292] | [0.0381] | [0.0293] | [0.0369] | [0.0289] | [0.0369] | [0.0293] | [0.0369] | [0.0294] |
| temperature (t-7) | -0.170*** | -0.189** |  |  |  |  |  |  |  |  |
|  | [0.0608] | [0.0886] |  |  |  |  |  |  |  |  |
| solar radiation (t-7) |  |  | -0.160*** | -0.0727 |  |  |  |  |  |  |
|  |  |  | [0.0573] | [0.0629] |  |  |  |  |  |  |
| humidity (t-7) |  |  |  |  | -0.0337 | -0.0687 |  |  |  |  |
|  |  |  |  |  | [0.0439] | [0.0649] |  |  |  |  |
| precipitation (t-7) |  |  |  |  |  |  | -0.0166 | -0.0414** |  |  |
|  |  |  |  |  |  |  | [0.0115] | [0.0180] |  |  |
| wind speed (t-7) |  |  |  |  |  |  |  |  | -0.0243 | -0.0998** |
|  |  |  |  |  |  |  |  |  | [0.0158] | [0.0468] |
| season index | 0.143** | 0.402*** | 0.154** | 0.401*** | 0.0911* | 0.425*** | 0.0790 | 0.407*** | 0.0777 | 0.412*** |
|  | [0.0556] | [0.0951] | [0.0654] | [0.0932] | [0.0486] | [0.0974] | [0.0498] | [0.0981] | [0.0499] | [0.0970] |
| Trend | Yes | Yes | Yes | Yes | Yes | Yes | Yes | Yes | Yes | Yes |
| Observations | 6,438 | 6,438 | 6,438 | 6,438 | 6,438 | 6,438 | 6,438 | 6,438 | 6,438 | 6,438 |
| Country | 37 | 37 | 37 | 37 | 37 | 37 | 37 | 37 | 37 | 37 |
| R-squared | 0.822 |  | 0.822 |  | 0.821 |  | 0.821 |  | 0.821 |  |

Note: the coefficients displayed are marginal effects. Standard errors (robust to within-country correlations for DFE) are reported in brackets. *** p < 0.01, ** < 0.05, * p < 0.1. *season index* is a qualitative variable accounting for meteorological seasons in the Northern and Southern hemispheres: = 1 for winter = 2 for spring, = 3 for summer, = 4 for autumn. For example, in our sample, the *season index* variable takes the value 1 (winter) for observations between January 1^st^ and 29^th^ February, 2020 in the Northern hemisphere and for observations between 1^st^ June and 27^th^ July, 2020 in the Southern hemisphere.

**J2. Direct effects of climate variables on Covid-19 fatalities: *season index***

|  | **Covid-19 fatalities** | | | | | | | | | |
| --- | --- | --- | --- | --- | --- | --- | --- | --- | --- | --- |
|  | DFE | MG | DFE | MG | DFE | MG | DFE | MG | DFE | MG |
|  | (1) | (2) | (3) | (4) | (5) | (6) | (7) | (8) | (9) | (10) |
| deathpop (t-1) | 0.589*** | 0.359*** | 0.593*** | 0.362*** | 0.590*** | 0.354*** | 0.593*** | 0.368*** | 0.593*** | 0.364*** |
|  | [0.111] | [0.0481] | [0.111] | [0.0483] | [0.111] | [0.0485] | [0.111] | [0.0480] | [0.111] | [0.0489] |
| deathpop (t-14) | 0.156*** | 0.112*** | 0.154*** | 0.107*** | 0.152*** | 0.102*** | 0.153*** | 0.107*** | 0.152*** | 0.107*** |
|  | [0.0463] | [0.0263] | [0.0472] | [0.0262] | [0.0458] | [0.0265] | [0.0465] | [0.0261] | [0.0459] | [0.0261] |
| casepop (t-14) | 0.00823** | 0.0181*** | 0.00863** | 0.0183*** | 0.00848** | 0.0177*** | 0.00867** | 0.0182*** | 0.00868** | 0.0184*** |
|  | [0.00337] | [0.00407] | [0.00343] | [0.00421] | [0.00341] | [0.00404] | [0.00344] | [0.00416] | [0.00343] | [0.00416] |
| temperature (t-14) | -0.0224*** | -0.0160* |  |  |  |  |  |  |  |  |
|  | [0.00593] | [0.00835] |  |  |  |  |  |  |  |  |
| solar radiation (t-14) |  |  | -0.00361 | 0.00508 |  |  |  |  |  |  |
|  |  |  | [0.00405] | [0.00539] |  |  |  |  |  |  |
| humidity (t-14) |  |  |  |  | -0.0163*** | -0.0189*** |  |  |  |  |
|  |  |  |  |  | [0.00549] | [0.00577] |  |  |  |  |
| precipitation (t-14) |  |  |  |  |  |  | 0.000359 | 0.000128 |  |  |
|  |  |  |  |  |  |  | [0.00269] | [0.00362] |  |  |
| wind speed (t-14) |  |  |  |  |  |  |  |  | -0.00304 | -0.00494 |
|  |  |  |  |  |  |  |  |  | [0.00288] | [0.0101] |
| season index | 0.00908** | 0.0138** | 0.00198 | 0.0106** | 0.00595 | 0.0119** | 0.000378 | 0.0129*** | 0.000146 | 0.0112** |
|  | [0.00416] | [0.00541] | [0.00462] | [0.00534] | [0.00424] | [0.00510] | [0.00454] | [0.00477] | [0.00472] | [0.00520] |
| Trend | Yes | Yes | Yes | Yes | Yes | Yes | Yes | Yes | Yes | Yes |
| Observations | 5,920 | 5,920 | 5,920 | 5,920 | 5,920 | 5,920 | 5,920 | 5,920 | 5,920 | 5,920 |
| Country | 37 | 37 | 37 | 37 | 37 | 37 | 37 | 37 | 37 | 37 |
| R-squared | 0.564 |  | 0.562 |  | 0.563 |  | 0.562 |  | 0.562 |  |

Note: the coefficients displayed are marginal effects. Standard errors (robust to within-country correlations for DFE) are reported in brackets. *** p < 0.01, ** < 0.05, * p < 0.1. *season index* is a qualitative variable accounting for meteorological seasons in the Northern and Southern hemispheres: = 1 for winter, = 2 for spring, = 3 for summer, = 4 for autumn.

**J3. Indirect effects of climate variables on Covid-19 infected cases through human mobility: *season index***

|  | **Covid-19 infected cases** | | | | | | | | | |
| --- | --- | --- | --- | --- | --- | --- | --- | --- | --- | --- |
|  | DFE | MG | DFE | MG | DFE | MG | DFE | MG | DFE | MG |
|  | (1) | (2) | (3) | (4) | (5) | (6) | (7) | (8) | (9) | (10) |
| casepop (t-1) | 0.695*** | 0.503*** | 0.691*** | 0.532*** | 0.699*** | 0.513*** | 0.701*** | 0.564*** | 0.701*** | 0.564*** |
|  | [0.0365] | [0.0404] | [0.0369] | [0.0358] | [0.0356] | [0.0408] | [0.0359] | [0.0356] | [0.0357] | [0.0357] |
| casepop (t-7) | 0.245*** | 0.232*** | 0.248*** | 0.253*** | 0.244*** | 0.229*** | 0.246*** | 0.246*** | 0.246*** | 0.250*** |
|  | [0.0380] | [0.0312] | [0.0384] | [0.0306] | [0.0376] | [0.0303] | [0.0374] | [0.0300] | [0.0375] | [0.0296] |
| mobility (t-7) | 6.16e-05 | -0.00294 | -0.00148* | -0.00538*** | -0.000196 | -0.00528*** | -5.73e-05 | -0.00324*** | -2.83e-05 | -0.00302** |
|  | [0.000740] | [0.00356] | [0.000796] | [0.00147] | [0.000583] | [0.00137] | [0.000496] | [0.00125] | [0.000559] | [0.00152] |
| temperature (t-7) | -0.320*** | -0.731*** |  |  |  |  |  |  |  |  |
|  | [0.0971] | [0.213] |  |  |  |  |  |  |  |  |
| temperature*mobility (t-7) | 0.00141 | 0.00607* |  |  |  |  |  |  |  |  |
|  | [0.000986] | [0.00317] |  |  |  |  |  |  |  |  |
| solar radiation (t-7) |  |  | -0.410*** | -0.587*** |  |  |  |  |  |  |
|  |  |  | [0.136] | [0.196] |  |  |  |  |  |  |
| solar radiation*mobility (t-7) |  |  | 0.00252** | 0.00617*** |  |  |  |  |  |  |
|  |  |  | [0.00109] | [0.00199] |  |  |  |  |  |  |
| humidity (t-7) |  |  |  |  | -0.147** | -0.728*** |  |  |  |  |
|  |  |  |  |  | [0.0694] | [0.140] |  |  |  |  |
| humidity*mobility (t-7) |  |  |  |  | 0.00114 | 0.00739*** |  |  |  |  |
|  |  |  |  |  | [0.000690] | [0.00134] |  |  |  |  |
| precipitation (t-7) |  |  |  |  |  |  | -0.0740 | -0.0672 |  |  |
|  |  |  |  |  |  |  | [0.0471] | [0.0539] |  |  |
| precipitation*mobility (t-7) |  |  |  |  |  |  | 0.000606 | 0.000105 |  |  |
|  |  |  |  |  |  |  | [0.000446] | [0.000582] |  |  |
| wind speed (t-7) |  |  |  |  |  |  |  |  | -0.0558 | 0.0515 |
|  |  |  |  |  |  |  |  |  | [0.0820] | [0.148] |
| wind speed*mobility (t-7) |  |  |  |  |  |  |  |  | 0.000334 | -0.00145 |
|  |  |  |  |  |  |  |  |  | [0.000719] | [0.00186] |
| season index | 0.135** | 0.338*** | 0.133** | 0.321*** | 0.0806 | 0.373*** | 0.0728 | 0.376*** | 0.0757 | 0.372*** |
|  | [0.0583] | [0.100] | [0.0649] | [0.0934] | [0.0502] | [0.104] | [0.0492] | [0.104] | [0.0507] | [0.102] |
| Trend | Yes | Yes | Yes | Yes | Yes | Yes | Yes | Yes | Yes | Yes |
| Observations | 6,364 | 6,364 | 6,364 | 6,364 | 6,364 | 6,364 | 6,364 | 6,364 | 6,364 | 6,364 |
| Country | 37 | 37 | 37 | 37 | 37 | 37 | 37 | 37 | 37 | 37 |
| R-squared | 0.822 |  | 0.822 |  | 0.821 |  | 0.821 |  | 0.821 |  |

Note: the coefficients displayed are marginal effects. Standard errors (robust to within-country correlations for DFE) are reported in brackets. *** p < 0.01, ** < 0.05, * p < 0.1. *season index* is a qualitative variable accounting for meteorological seasons in the Northern and Southern hemispheres: = 1 for winter, = 2 for spring, = 3 for summer, = 4 for autumn.

**J4. Indirect effects of climate variables on Covid-19 fatalities through human mobility: *season index***

|  | **Covid-19 fatalities** | | | | | | | | | |
| --- | --- | --- | --- | --- | --- | --- | --- | --- | --- | --- |
|  | DFE | MG | DFE | MG | DFE | MG | DFE | MG | DFE | MG |
|  | (1) | (2) | (3) | (4) | (5) | (6) | (7) | (8) | (9) | (10) |
| deathpop (t-1) | 0.601*** | 0.245*** | 0.603*** | 0.270*** | 0.602*** | 0.246*** | 0.612*** | 0.283*** | 0.610*** | 0.281*** |
|  | [0.120] | [0.0439] | [0.121] | [0.0449] | [0.120] | [0.0446] | [0.120] | [0.0460] | [0.121] | [0.0488] |
| deathpop (t-14) | 0.145*** | 0.0888*** | 0.142*** | 0.0718*** | 0.137*** | 0.0796*** | 0.131** | 0.0594** | 0.131** | 0.0591** |
|  | [0.0494] | [0.0272] | [0.0506] | [0.0277] | [0.0489] | [0.0274] | [0.0493] | [0.0277] | [0.0490] | [0.0274] |
| casepop (t-14) | 0.00536* | 0.0111*** | 0.00588** | 0.0140*** | 0.00581* | 0.0109*** | 0.00706** | 0.0143*** | 0.00699** | 0.0145*** |
|  | [0.00295] | [0.00357] | [0.00275] | [0.00415] | [0.00299] | [0.00355] | [0.00302] | [0.00411] | [0.00305] | [0.00422] |
| mobility (t-14) | -0.000531** | -0.00119*** | -0.000672*** | -0.00117*** | -0.000495** | -0.00113*** | -0.000450** | -0.000885*** | -0.000459*** | -0.000917*** |
|  | [0.000206] | [0.000324] | [0.000239] | [0.000276] | [0.000187] | [0.000230] | [0.000167] | [0.000288] | [0.000168] | [0.000245] |
| temperature (t-14) | -0.0645*** | -0.0915*** |  |  |  |  |  |  |  |  |
|  | [0.0188] | [0.0292] |  |  |  |  |  |  |  |  |
| temperature*mobility (t-14) | 0.000503*** | 0.000980*** |  |  |  |  |  |  |  |  |
|  | [0.000177] | [0.000308] |  |  |  |  |  |  |  |  |
| solar radiation (t-14) |  |  | -0.0608*** | -0.0546*** |  |  |  |  |  |  |
|  |  |  | [0.0203] | [0.0194] |  |  |  |  |  |  |
| solar radiation*mobility (t-14) |  |  | 0.000558*** | 0.000702*** |  |  |  |  |  |  |
|  |  |  | [0.000202] | [0.000262] |  |  |  |  |  |  |
| humidity (t-14) |  |  |  |  | -0.0519*** | -0.0995*** |  |  |  |  |
|  |  |  |  |  | [0.0165] | [0.0241] |  |  |  |  |
| humidity*mobility (t-14) |  |  |  |  | 0.000450*** | 0.000993*** |  |  |  |  |
|  |  |  |  |  | [0.000156] | [0.000244] |  |  |  |  |
| precipitation (t-14) |  |  |  |  |  |  | 0.00184 | -0.0257 |  |  |
|  |  |  |  |  |  |  | [0.00937] | [0.0166] |  |  |
| precipitation*mobility (t-14) |  |  |  |  |  |  | 5.73e-07 | 0.000458 |  |  |
|  |  |  |  |  |  |  | [7.30e-05] | [0.000375] |  |  |
| wind speed (t-14) |  |  |  |  |  |  |  |  | 0.00604 | -0.00249 |
|  |  |  |  |  |  |  |  |  | [0.00954] | [0.0163] |
| wind speed*mobility (t-14) |  |  |  |  |  |  |  |  | -0.000104 | -3.46e-05 |
|  |  |  |  |  |  |  |  |  | [9.10e-05] | [0.000113] |
| season index | 0.00535 | 0.0255** | 0.00140 | 0.0135** | 0.00232 | 0.0238*** | 0.00126 | 0.0164** | 0.000397 | 0.0154** |
|  | [0.00522] | [0.0101] | [0.00548] | [0.00641] | [0.00559] | [0.00845] | [0.00474] | [0.00642] | [0.00524] | [0.00681] |
| Trend | Yes | Yes | Yes | Yes | Yes | Yes | Yes | Yes | Yes | Yes |
| Observations | 5,846 | 5,846 | 5,846 | 5,846 | 5,846 | 5,846 | 5,846 | 5,846 | 5,846 | 5,846 |
| Country | 37 | 37 | 37 | 37 | 37 | 37 | 37 | 37 | 37 | 37 |
| R-squared | 0.600 |  | 0.600 |  | 0.599 |  | 0.597 |  | 0.597 |  |

Note: the coefficients displayed are marginal effects. Standard errors (robust to within-country correlations for DFE) are reported in brackets. *** p < 0.01, ** < 0.05, * p < 0.1. *season index* is a qualitative variable accounting for meteorological seasons in the Northern and Southern hemispheres: = 1 for winter, = 2 for spring, = 3 for summer, = 4 for autumn.

**J5. Direct effects of climate variables on Covid-19 infected cases: rolling-window mean of climate variables**

|  | **Covid-19 infected cases** | | | | | | | | | |
| --- | --- | --- | --- | --- | --- | --- | --- | --- | --- | --- |
|  | DFE | MG | DFE | MG | DFE | MG | DFE | MG | DFE | MG |
|  | (1) | (2) | (3) | (4) | (5) | (6) | (7) | (8) | (9) | (10) |
| casepop (t-1) | 0.699*** | 0.588*** | 0.694*** | 0.587*** | 0.701*** | 0.593*** | 0.700*** | 0.591*** | 0.696*** | 0.602*** |
|  | [0.0358] | [0.0349] | [0.0364] | [0.0334] | [0.0359] | [0.0350] | [0.0363] | [0.0331] | [0.0377] | [0.0321] |
| casepop (t-7) | 0.242*** | 0.248*** | 0.244*** | 0.272*** | 0.245*** | 0.250*** | 0.245*** | 0.277*** | 0.247*** | 0.269*** |
|  | [0.0364] | [0.0301] | [0.0361] | [0.0310] | [0.0365] | [0.0299] | [0.0363] | [0.0305] | [0.0367] | [0.0303] |
| temperature (t-7) | -0.115* | -0.150** |  |  |  |  |  |  |  |  |
|  | [0.0594] | [0.0698] |  |  |  |  |  |  |  |  |
| solar radiation (t-7) |  |  | 0.00497 | 0.0359 |  |  |  |  |  |  |
|  |  |  | [0.0384] | [0.0371] |  |  |  |  |  |  |
| humidity (t-7) |  |  |  |  | -0.0398 | -0.0779 |  |  |  |  |
|  |  |  |  |  | [0.0434] | [0.0530] |  |  |  |  |
| precipitation (t-7) |  |  |  |  |  |  | -0.0316* | -0.0363* |  |  |
|  |  |  |  |  |  |  | [0.0158] | [0.0188] |  |  |
| wind speed (t-7) |  |  |  |  |  |  |  |  | -0.0719*** | -0.0916*** |
|  |  |  |  |  |  |  |  |  | [0.0218] | [0.0314] |
| mean climate var. (t-7) | 0.0182 | -0.480 | -0.223** | -0.765*** | 0.0725 | 0.0659 | 0.129 | -0.0724 | 0.373** | 0.163 |
|  | [0.0870] | [0.456] | [0.0832] | [0.287] | [0.0697] | [0.323] | [0.135] | [0.263] | [0.137] | [0.371] |
| Trend | Yes | Yes | Yes | Yes | Yes | Yes | Yes | Yes | Yes | Yes |
| Observations | 6,253 | 6,253 | 6,253 | 6,253 | 6,253 | 6,253 | 6,253 | 6,253 | 6,253 | 6,253 |
| Country | 37 | 37 | 37 | 37 | 37 | 37 | 37 | 37 | 37 | 37 |
| R-squared | 0.818 |  | 0.819 |  | 0.818 |  | 0.818 |  | 0.819 |  |

Note: the coefficients displayed are marginal effects. Standard errors (robust to within-country correlations for DFE) are reported in brackets. *** p < 0.01, ** < 0.05, * p < 0.1. *mean climate var.* corresponds to the seven-days lag of the mean of each climate variable computed on a 30-days rolling-window.

**J6. Direct effects of climate variables on Covid-19 fatalities: rolling-window mean of climate variables**

|  | **Covid-19 fatalities** | | | | | | | | | |
| --- | --- | --- | --- | --- | --- | --- | --- | --- | --- | --- |
|  | DFE | MG | DFE | MG | DFE | MG | DFE | MG | DFE | MG |
|  | (1) | (2) | (3) | (4) | (5) | (6) | (7) | (8) | (9) | (10) |
| deathpop (t-1) | 0.590*** | 0.327*** | 0.589*** | 0.349*** | 0.590*** | 0.333*** | 0.590*** | 0.355*** | 0.584*** | 0.349*** |
|  | [0.112] | [0.0480] | [0.110] | [0.0473] | [0.112] | [0.0493] | [0.109] | [0.0468] | [0.109] | [0.0483] |
| deathpop (t-14) | 0.154*** | 0.0733*** | 0.159*** | 0.107*** | 0.152*** | 0.0676*** | 0.160*** | 0.0992*** | 0.163*** | 0.103*** |
|  | [0.0459] | [0.0261] | [0.0478] | [0.0267] | [0.0452] | [0.0256] | [0.0466] | [0.0268] | [0.0459] | [0.0252] |
| casepop (t-14) | 0.00797** | 0.0164*** | 0.00792** | 0.0196*** | 0.00833** | 0.0171*** | 0.00861** | 0.0189*** | 0.00850** | 0.0191*** |
|  | [0.00325] | [0.00390] | [0.00333] | [0.00406] | [0.00330] | [0.00394] | [0.00342] | [0.00405] | [0.00349] | [0.00395] |
| temperature (t-14) | -0.0135*** | -0.00840 |  |  |  |  |  |  |  |  |
|  | [0.00429] | [0.00668] |  |  |  |  |  |  |  |  |
| solar radiation (t-14) |  |  | 0.0125** | 0.00744* |  |  |  |  |  |  |
|  |  |  | [0.00571] | [0.00410] |  |  |  |  |  |  |
| humidity (t-14) |  |  |  |  | -0.0155*** | -0.0128** |  |  |  |  |
|  |  |  |  |  | [0.00477] | [0.00541] |  |  |  |  |
| precipitation (t-14) |  |  |  |  |  |  | -0.00259 | 0.000102 |  |  |
|  |  |  |  |  |  |  | [0.00297] | [0.00374] |  |  |
| wind speed (t-14) |  |  |  |  |  |  |  |  | -0.00844** | -0.00921 |
|  |  |  |  |  |  |  |  |  | [0.00349] | [0.0117] |
| mean climate var. (t-14) | -0.00775 | -0.140*** | -0.0288*** | 0.00991 | 0.00244 | -0.110** | 0.0292 | 0.0101 | 0.0528*** | 0.103*** |
|  | [0.00868] | [0.0376] | [0.00804] | [0.0302] | [0.00843] | [0.0432] | [0.0176] | [0.0121] | [0.0153] | [0.0398] |
| Trend | Yes | Yes | Yes | Yes | Yes | Yes | Yes | Yes | Yes | Yes |
| Observations | 5,920 | 5,920 | 5,920 | 5,920 | 5,920 | 5,920 | 5,920 | 5,920 | 5,920 | 5,920 |
| Country | 37 | 37 | 37 | 37 | 37 | 37 | 37 | 37 | 37 | 37 |
| R-squared | 0.563 |  | 0.564 |  | 0.563 |  | 0.563 |  | 0.565 |  |

Note: the coefficients displayed are marginal effects. Standard errors (robust to within-country correlations for DFE) are reported in brackets. *** p < 0.01, ** < 0.05, * p < 0.1. *mean climate var.* corresponds to the seven-days lag of the mean of each climate variable computed on a 30-days rolling-window.

**J7. Indirect effects of climate variables on Covid-19 infected cases through human mobility: rolling-window mean of climate variables**

|  | **Covid-19 infected cases** | | | | | | | | | |
| --- | --- | --- | --- | --- | --- | --- | --- | --- | --- | --- |
|  | DFE | MG | DFE | MG | DFE | MG | DFE | MG | DFE | MG |
|  | (1) | (2) | (3) | (4) | (5) | (6) | (7) | (8) | (9) | (10) |
| casepop (t-1) | 0.696*** | 0.495*** | 0.688*** | 0.468*** | 0.698*** | 0.502*** | 0.700*** | 0.520*** | 0.696*** | 0.543*** |
|  | [0.0358] | [0.0410] | [0.0367] | [0.0378] | [0.0357] | [0.0414] | [0.0363] | [0.0364] | [0.0374] | [0.0359] |
| casepop (t-7) | 0.240*** | 0.220*** | 0.242*** | 0.245*** | 0.242*** | 0.213*** | 0.244*** | 0.256*** | 0.248*** | 0.247*** |
|  | [0.0365] | [0.0321] | [0.0366] | [0.0325] | [0.0364] | [0.0306] | [0.0364] | [0.0310] | [0.0372] | [0.0297] |
| mobility (t-7) | -0.000205 | -0.00192 | -0.00155* | -0.0142*** | -0.000566 | -0.00751*** | -0.000158 | -0.00683*** | 0.000230 | -0.00378** |
|  | [0.000672] | [0.00343] | [0.000877] | [0.00364] | [0.000582] | [0.00221] | [0.000584] | [0.00164] | [0.000612] | [0.00179] |
| temperature (t-7) | -0.281** | -0.800*** |  |  |  |  |  |  |  |  |
|  | [0.105] | [0.220] |  |  |  |  |  |  |  |  |
| temperature*mobility (t-7) | 0.00179* | 0.00726** |  |  |  |  |  |  |  |  |
|  | [0.000993] | [0.00301] |  |  |  |  |  |  |  |  |
| solar radiation (t-7) |  |  | -0.301** | -0.292* |  |  |  |  |  |  |
|  |  |  | [0.131] | [0.173] |  |  |  |  |  |  |
| solar radiation*mobility (t-7) |  |  | 0.00308** | 0.00430** |  |  |  |  |  |  |
|  |  |  | [0.00119] | [0.00195] |  |  |  |  |  |  |
| humidity (t-7) |  |  |  |  | -0.167** | -0.667*** |  |  |  |  |
|  |  |  |  |  | [0.0701] | [0.129] |  |  |  |  |
| humidity*mobility (t-7) |  |  |  |  | 0.00132* | 0.00649*** |  |  |  |  |
|  |  |  |  |  | [0.000685] | [0.00122] |  |  |  |  |
| precipitation (t-7) |  |  |  |  |  |  | -0.0952* | -0.0668 |  |  |
|  |  |  |  |  |  |  | [0.0489] | [0.0507] |  |  |
| precipitation*mobility (t-7) |  |  |  |  |  |  | 0.000671 | 1.42e-05 |  |  |
|  |  |  |  |  |  |  | [0.000471] | [0.000572] |  |  |
| wind speed (t-7) |  |  |  |  |  |  |  |  | -0.0962 | 0.0358 |
|  |  |  |  |  |  |  |  |  | [0.0871] | [0.121] |
| wind speed*mobility (t-7) |  |  |  |  |  |  |  |  | 0.000253 | -0.00119 |
|  |  |  |  |  |  |  |  |  | [0.000746] | [0.00157] |
| mean climate var. (t-7) | -0.0208 | -0.399 | -0.233*** | -1.592*** | 0.0686 | 0.221 | 0.131 | 0.180 | 0.375** | -0.00456 |
|  | [0.0852] | [0.484] | [0.0839] | [0.531] | [0.0768] | [0.467] | [0.140] | [0.336] | [0.138] | [0.529] |
| Trend | Yes | Yes | Yes | Yes | Yes | Yes | Yes | Yes | Yes | Yes |
| Observations | 6,179 | 6,179 | 6,179 | 6,179 | 6,179 | 6,179 | 6,179 | 6,179 | 6,179 | 6,179 |
| Country | 37 | 37 | 37 | 37 | 37 | 37 | 37 | 37 | 37 | 37 |
| R-squared | 0.818 |  | 0.819 |  | 0.818 |  | 0.818 |  | 0.819 |  |

Note: the coefficients displayed are marginal effects. Standard errors (robust to within-country correlations for DFE) are reported in brackets. *** p < 0.01, ** < 0.05, * p < 0.1. *mean climate var.* corresponds to the seven-days lag of the mean of each climate variable computed on a 30-days rolling-window.

**J8. Indirect effects of climate variables on Covid-19 fatalities through human mobility: rolling-window mean of climate variables**

|  | **Covid-19 fatalities** | | | | | | | | | |
| --- | --- | --- | --- | --- | --- | --- | --- | --- | --- | --- |
|  | DFE | MG | DFE | MG | DFE | MG | DFE | MG | DFE | MG |
|  | (1) | (2) | (3) | (4) | (5) | (6) | (7) | (8) | (9) | (10) |
| deathpop (t-1) | 0.601*** | 0.233*** | 0.598*** | 0.232*** | 0.601*** | 0.239*** | 0.607*** | 0.266*** | 0.603*** | 0.265*** |
|  | [0.120] | [0.0445] | [0.120] | [0.0409] | [0.120] | [0.0451] | [0.118] | [0.0438] | [0.119] | [0.0474] |
| deathpop (t-14) | 0.144*** | 0.0714*** | 0.148*** | 0.0961*** | 0.138*** | 0.0652** | 0.138** | 0.0806*** | 0.141*** | 0.0641** |
|  | [0.0497] | [0.0268] | [0.0518] | [0.0281] | [0.0491] | [0.0261] | [0.0509] | [0.0268] | [0.0498] | [0.0268] |
| casepop (t-14) | 0.00521* | 0.0106*** | 0.00516* | 0.0144*** | 0.00576* | 0.0107*** | 0.00688** | 0.0148*** | 0.00690** | 0.0149*** |
|  | [0.00291] | [0.00352] | [0.00269] | [0.00372] | [0.00303] | [0.00351] | [0.00299] | [0.00388] | [0.00312] | [0.00397] |
| mobility (t-14) | -0.000538** | -0.000981*** | -0.000679*** | -0.00171*** | -0.000535*** | -0.00118*** | -0.000493*** | -0.00115*** | -0.000424** | -0.000982*** |
|  | [0.000201] | [0.000282] | [0.000243] | [0.000380] | [0.000185] | [0.000248] | [0.000180] | [0.000366] | [0.000172] | [0.000298] |
| temperature (t-14) | -0.0614*** | -0.0958*** |  |  |  |  |  |  |  |  |
|  | [0.0178] | [0.0247] |  |  |  |  |  |  |  |  |
| temperature*mobility (t-14) | 0.000510*** | 0.00104*** |  |  |  |  |  |  |  |  |
|  | [0.000182] | [0.000261] |  |  |  |  |  |  |  |  |
| solar radiation (t-14) |  |  | -0.0478** | -0.0394** |  |  |  |  |  |  |
|  |  |  | [0.0187] | [0.0162] |  |  |  |  |  |  |
| solar radiation*mobility (t-14) |  |  | 0.000587*** | 0.000661*** |  |  |  |  |  |  |
|  |  |  | [0.000207] | [0.000249] |  |  |  |  |  |  |
| humidity (t-14) |  |  |  |  | -0.0558*** | -0.0878*** |  |  |  |  |
|  |  |  |  |  | [0.0175] | [0.0225] |  |  |  |  |
| humidity*mobility (t-14) |  |  |  |  | 0.000439*** | 0.000826*** |  |  |  |  |
|  |  |  |  |  | [0.000150] | [0.000205] |  |  |  |  |
| precipitation (t-14) |  |  |  |  |  |  | -0.00181 | -0.0250 |  |  |
|  |  |  |  |  |  |  | [0.00918] | [0.0165] |  |  |
| precipitation*mobility (t-14) |  |  |  |  |  |  | 3.59e-06 | 0.000439 |  |  |
|  |  |  |  |  |  |  | [7.24e-05] | [0.000385] |  |  |
| wind speed (t-14) |  |  |  |  |  |  |  |  | 0.00204 | -0.0118 |
|  |  |  |  |  |  |  |  |  | [0.00889] | [0.0216] |
| wind speed*mobility (t-14) |  |  |  |  |  |  |  |  | -0.000113 | 7.70e-05 |
|  |  |  |  |  |  |  |  |  | [0.000100] | [0.000182] |
| mean climate var. (t-14) | -0.00153 | -0.0784** | -0.0286*** | -0.0605 | 0.0126 | -0.0480 | 0.0332 | 0.0251 | 0.0462*** | 0.0844*** |
|  | [0.00858] | [0.0328] | [0.00980] | [0.0389] | [0.00826] | [0.0392] | [0.0199] | [0.0224] | [0.0164] | [0.0291] |
| Trend | Yes | Yes | Yes | Yes | Yes | Yes | Yes | Yes | Yes | Yes |
| Observations | 5,846 | 5,846 | 5,846 | 5,846 | 5,846 | 5,846 | 5,846 | 5,846 | 5,846 | 5,846 |
| Country | 37 | 37 | 37 | 37 | 37 | 37 | 37 | 37 | 37 | 37 |
| R-squared | 0.600 |  | 0.601 |  | 0.599 |  | 0.598 |  | 0.599 |  |

Note: the coefficients displayed are marginal effects. Standard errors (robust to within-country correlations for DFE) are reported in brackets. *** p < 0.01, ** < 0.05, * p < 0.1. *mean climate var.* corresponds to the seven-days lag of the mean of each climate variable computed on a 30-days rolling-window.
